# The Tree versus the Forest: The Fungal Tree of Life and the Topological Diversity within the Yeast Phylome

**DOI:** 10.1371/journal.pone.0004357

**Published:** 2009-02-03

**Authors:** Marina Marcet-Houben, Toni Gabaldón

**Affiliations:** Bioinformatics Department, Centro de Investigación Príncipe Felipe, València, Spain.; Institut Pasteur, France

## Abstract

A recurrent topic in phylogenomics is the combination of various sequence alignments to reconstruct a tree that describes the evolutionary relationships within a group of species. However, such approach has been criticized for not being able to properly represent the topological diversity found among gene trees. To evaluate the representativeness of species trees based on concatenated alignments, we reconstruct several fungal species trees and compare them with the complete collection of phylogenies of genes encoded in the *Saccharomyces cerevisiae* genome. We found that, despite high levels of among-gene topological variation, the species trees do represent widely supported phylogenetic relationships. Most topological discrepancies between gene and species trees are concentrated in certain conflicting nodes. We propose to map such information on the species tree so that it accounts for the levels of congruence across the genome. We identified the lack of sufficient accuracy of current alignment and phylogenetic methods as an important source for the topological diversity encountered among gene trees. Finally, we discuss the implications of the high levels of topological variation for phylogeny-based orthology prediction strategies.

## Introduction

The advent of the genome era and the availability of a growing number of fully-sequenced genomes have changed the way in which biologists study the evolutionary relationships among groups of organisms. For instance, the use of phylogenetics in the context of whole genomes, a field known as phylogenomics [1], allows for the combination of evolutionary signals from various genes into a single tree. It has long been observed that phylogenetic trees built from different genes may provide conflicting topologies. Thus, the use of multiple gene approaches is a way to average out these discrepancies in order to provide a single topology that is expected to reflect the true evolutionary relationships more accurately. In recent years, the use of multi-gene approaches, and especially gene concatenation, is becoming the method of choice in most studies aiming to elucidate the evolutionary relationships among a group of species [2]. Such approaches are, however, not free from criticism. For instance, it has been argued that they use the information derived from a small fraction of the genes in a genome and, therefore, cannot represent the actual diversity of evolutionary histories within a genome [3]. Indeed, initial genome-wide phylogenetic studies have shown that the topological diversity encountered across a genome is high [4], [5]. Besides questioning the validity of species trees, these findings have raised doubts regarding the possible sources for the high topological variability and the implications for large-scale phylogenetic inferences such as the prediction of orthology relationships.

Here we address the question of whether species trees constructed with standard alignment concatenation approaches do fairly represent the topologies that can be found in gene phylogenies across a genome. Conversely, we test whether the topological information found across all genes in a genome can be used to identify conflicting nodes and provide alternative reliability values in species trees. We test these ideas by using molecular data from fungal genomes, the group of eukaryotic organisms that is best sampled in terms of fully sequenced genomes [6]. Currently, more than 60 fungal species have been sequenced, including many human pathogens as well as other species of industrial or agricultural interest. This has facilitated that the evolutionary relationships among fungi have been addressed by means of phylogenomic methods, being gene concatenation the most widely used [7]–[9]. To assess the extent of congruence between trees based on concatenated alignments and individual phylogenies, we compare the topology of phylogenies of genes encoded in the yeast genome with fungal species trees reconstructed from the concatenated alignments of widespread proteins present across different sets of fungal species. Our results show that, despite the large topological diversity of the yeast phylome, most nodes in the species tree do represent genome-wide supported evolutionary relationships. Some conflicting nodes, however, concentrate most of the topological variations found between gene and species trees. We propose to incorporate such information in the tree of life in the form of genome-wide levels of topological support, thereby identifying conflicting nodes. Finally, some of the possible causes for the existing topological diversity within a genome and its implications for orthology prediction are discussed.

## Results and Discussion

### Growing the fungal species tree

Recently, several groups have proposed fungal species trees based on the concatenated alignment of proteins selected from fully-sequenced genomes [7]–[11]. The various studies considered different sets of species but used a similar method to select genes that were single-copy and widespread in their respective sets. A natural consequence of this methodology is that the number of genes considered in the phylogenetic analysis diminishes as the number of genomes included grows. In this way, the study of Robbertse et al [9], limited to 17 ascomycota species, comprised 781 protein sequences (195,664 positions) in the alignment, whereas those of Kuramae et al [7] and Fitzpatrick et al [8], included, respectively, 531 genes (67,101 positions) for 24 species and 153 genes (38,000 positions) for 42 species. Remarkably, all these phylogenies are largely similar, at least for the set of species that they all have in common. Exceptions to this overall agreement include the phylogenetic position of *Stagonospora nodorum*, the relative branching order of *Candida glabrata* and *Saccharomyces castellii*, and some relative positions within the *Candida* genus.

**Figure 1:**
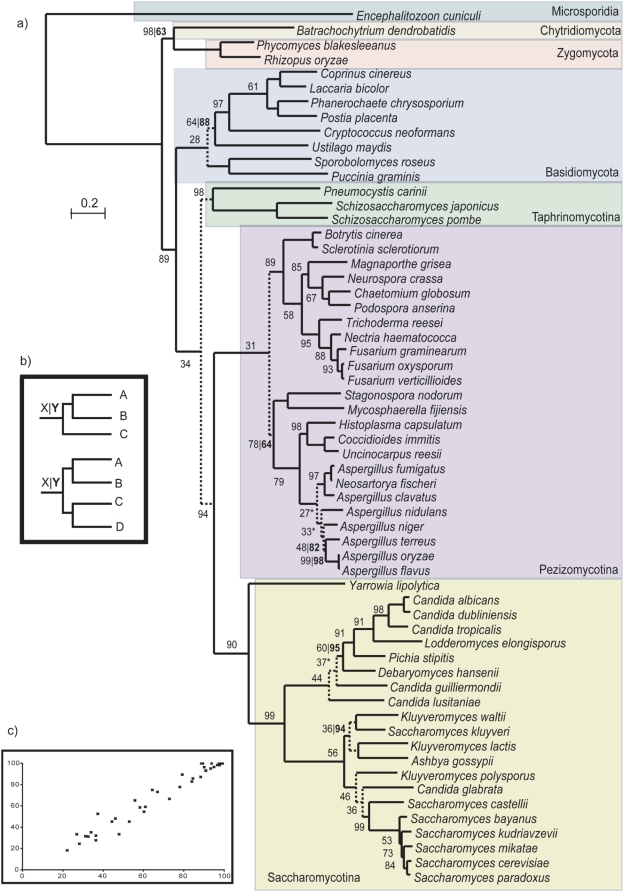
The Fungal species tree. A) Phylogenetic tree representing the evolutionary relationships among the 60 fungal species considered in the study, as resulting from the ML analysis of the concatenated alignment of 69 widespread proteins. Numbers on the nodes indicate two different types of support values. The first number indicates the phylome support for that node, that is, the percentage of trees in the phylome that support the specific arrangement of the three or four groups of species defined by its daughter nodes (see B). An asterisk next to this number indicates that the topology obtained by the species tree is not the most common among the trees in the phylome. Whenever there is a second number (in bold), this indicates the bootstrap support when this is lower than 100. Partitions that do not have this second number have a bootstrap support of 100. Branches with dashed lines indicate evolutionary relationships that are supported by less than 50% of the trees in the phylome. B) Schematic representation of the two types of support values for the different nodes in the tree. X indicates the phylome support for the specific topology indicated by that node. Two types of nodes do exist attending to the number of partitions delimited by their daughter nodes. A first class of nodes (top), delimit relative topologies of three partitions (A, B and C), whereas a second class (bottom) delimit four partitions (A, B, C and D). Phylome support values indicate the percentage of trees that show exactly the relative grouping of the three or four groups delimited by the node. This percentage is expressed over the fraction of trees that contain at least one species from each of the partitions considered. The second number (Y) indicates the bootstrap support for the partition delimited by that node, but does not provide specific support for the specific arrangement of the sub-partitions within that partition. C) Correlation between the fungal species tree topologies recovered by the individual trees included in the concatenated alignment (Y axis) and all the trees in the phylome (X axis). In both cases the fraction of trees that are compatible with a given topology, as computed with the topology scanning algorithm, is represented.

We used a similar approach to reconstruct a broader fungal species tree including 60 fungi with completely-sequenced genomes (see supplementary table S1). To achieve this, we built a concatenated alignment of 69 widespread proteins that were present in at least 58 of the 60 species used and displayed one to one orthology relationships (see Material and Methods). The removal of positions with gaps in more than 50% of the sequences resulted in a trimmed alignment of 31,123 amino acid positions, which was subsequently used for Maximum Likelihood (ML) pylogenetic reconstruction, using a 4-rates gamma distribution model. Figure 1 shows the resulting tree, which is fairly congruent with previous fungal species trees. Additionally, and to investigate the possible effects that the taxonomic sampling and the number of sequences involved may have in the final topology, we reconstructed three more species trees based on different sets of species. First, a well-sampled tree focusing on the 21 species from the Saccharomycotina group was built from a concatenated alignment of 1,137 widespread proteins. Next, another tree was built from the concatenation of 2007 proteins from the 12 species that belong to the *Saccharomyces* genus. Finally, a tree with the same number of species but each one sampled from the main fungal clades, was built using 217 concatenated alignments (see methods). The list of proteins included in each species tree is provided in the supplementary material (supplementary table S4). We will refer to these fungal species trees as T60, T21, T12a and T12b, respectively. No major differences were encountered in terms of the relative topologies for the species they have in common between the different trees (see supplementary material figures S1, S2 and S3).

### One tree fits all? : pattern pluralism within the yeast phylome

Many authors interpret the high level of similarity among different species trees as an indication that the proposed phylogeny reflects the real evolutionary relationships of the species included. A question that remains under discussion, however, is how well this tree represents the topological diversity encountered among trees from all the genes encoded in a genome. To evaluate this, we reconstructed the complete collection of phylogenies of the genes encoded in the *S. cerevisiae* genome, that is, the yeast phylome. To do so, we applied a similar pipeline as the one used to reconstruct the human phylome [4] (see methods). We derived four versions of the yeast phylome that differ in their taxonomic scope and correspond to the species samples used in the species trees described earlier. The resulting 111,760 phylogenies and 22,352 alignments have been deposited in PhylomeDB [12] (http://www.phylomedb.org; phylome codes SceP60, SceP21, SceP12a and SceP12b)

Some methodologies have been previously proposed to explicitly address the issue of concordance between species trees and individual gene trees [13], [14]. Such approaches have been successfully applied to compare species trees of eight fungal species with the corresponding 106 phylogenies of widespread single-copy genes. However, these methods cannot account for gene phylogenies that include gene loss and duplication events and are not feasible for large datasets as the ones considered here. Here, we use a simple measure of concordance, which consists of evaluating whether the topology of each single gene tree is fully compatible with that proposed by the species tree (see methods). Our results show that most individual gene phylogenies contain incompatibilities with the species tree. Of the 5804 trees of the phylome, only 410 (7.1%) were fully compatible with the topology in T60. Similar levels of congruence were observed for T21 (7.5%), whereas T12a showed a slight increase (12.3%). A marked improvement was observed in T12b (30.1%), suggesting that this set of distantly related species can be better resolved. These differences in congruence levels were generally similar when only partitions with high supports were considered (see supplementary figure S4).

It must be noted that our measure for topological consistency (full compatibility) is highly stringent, since a single mismatch would render two trees inconsistent. Interestingly, when the consistency is evaluated for each single node in the species tree a different picture does emerge. Indeed, when the level of topological congruence is expressed for each specific internal node of the proposed species tree (figure 1), the result is a tree where most of the nodes (73%) show the topology that is most represented (>50%) among the trees in the phylome. Several conflicting nodes, in contrast, are supported by smaller percentages of the trees in the phylome. In three nodes the topology found by the tree of life is not even the most represented among the trees in the phylome (see figure 1 and supplementary table S2). Nodes with low representation in the phylome do not always correspond to partitions that have low bootstrap values, indicating that bootstrap support in phylogenomic analyses can be misleading. These discrepancies cannot be explained by a topological bias in the sample used to reconstruct the species tree, since there is a high correlation between the topologies in the nodes of the sampled trees and that of the entire phylome (figure 1C). We conclude from this analysis that, despite the high topological variation, species trees reconstructed from concatenated alignments do represent, at least for most of their nodes, the strongest phylogenetic signals observed along a genome. However, to properly reflect that some of the topologies are not widely supported by the majority of gene trees, we propose that these should be indicated by dashed lines. A reasonable cut-off could be set at 50%, as shown in figure 1. A more conservative decision could consist of collapsing these branches with low support, thereby introducing some polytomies. This will provide a less resolved species tree in which only dichotomies supported by a majority of the gene trees are shown.

Additionally, these under-represented nodes seem to correspond to topologies that are less robust to variations in taxonomic sampling. To assess this, we reconstructed nine additional species trees using randomly-chosen sets of 50, 40 and 30 species from our set (see supplementary table S3). Combinations that did not contained the species *S. cerevisiae* and did not provided a set of at least 30 widespread proteins for the concatenation were discarded. In each case the tree was reconstructed from the concatenated alignment of the proteins that were widespread in the specific species sample. The three species trees with 30 species were fully congruent with T60. The remaining six trees did present slight topological variations in relation to T60 that mostly affected nodes with low support in the phylome (see supplementary material figure S5). Of the 16 topological discrepancies with T60 found in these alternative trees, 13(81%) affected nodes with support lower than 50%. The relative placement of *Debaromyces hansenii* and *Aspergillus nidulans* within their respective groups and the position of Dothideomycetes species within the Pezizomycotina were the evolutionary relationships that were most affected by the taxonomic sampling.

### Implications for phylogeny-based orthology prediction

**Figure 2:**
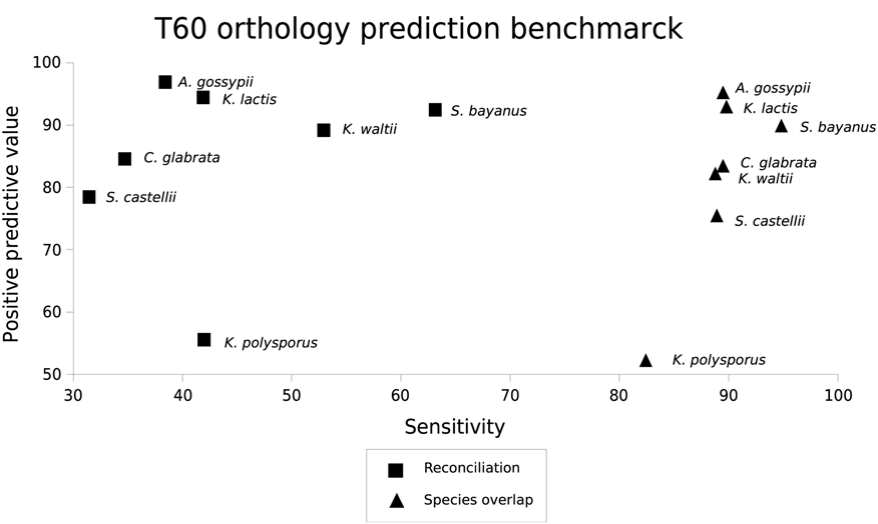
Comparison of different orthology inference algorithms. The synteny based and manually curated orthology predictions available at YGOB database [18] is taken as a golden set to compute the number of true positives (TP), false positives (FP) and false negatives (FN) yielded by each method. For each method, the sensitivity S = TP/(TP+FN) and the positive predictive value P = TP/(TP+FP) are computed.

Besides the reconstruction of species phylogenies, the existing high degree of topological variability in genome-wide data is likely to affect other applications of large-scale phylogenetic analyses. One of such applications is the large scale inference of phylogeny-based orthology predictions [12], [15], [16]. Such phylogeny-based methods are being increasingly used and are considered more accurate than standard pair-wise based methodologies [16]. There are two main approaches to infer orthology relationships from phylogenetic trees, namely reconciliation with the species tree [17] and the use of species overlap information to ascertain whether a node represents a duplication or speciation event [4]. We previously suggested that species-overlap algorithms would be more appropriate to cope with the topological diversity in single-gene phylogenies [4]. To test this, we applied both a strict tree reconciliation method and our previously described species-overlap algorithm to predict orthology relationships of all yeast genes. The orthology predictions from both methods were compared with the high-quality synteny-based orthology predictions from YGOB [18]. Although we observed no major differences in terms of positive predictive values between the two methods, there is a significant increase in terms of sensitivity when the species overlap algorithm is used (figure 2). This algorithm correctly predicted 82–96% of the true orthology relationships as compared to 32–65% values reached by species reconciliation, indicating that a relaxed consideration of tree topology is more appropriate.

### Lack of sufficient accuracy of current phylogenetic methods might explain a significant part of the topological diversity

Finally, we investigated some of the possible sources for the high topological variability observed. In principle, two main causes may be envisaged. First, some evolutionary processes such as horizontal gene transfer or gene duplication followed by differential gene loss may result in a divergent gene tree topology as compared to the actual species phylogeny. Alternatively, the topological variation might just be the result of insufficient accuracy of the methodology used. Two recent studies support the latter hypothesis by showing that different alignment reconstruction methods often result in different topologies [19] and that trees reconstructed from longer alignments are more likely to conform to the species tree [5]. In our case, we did not observe significant differences in terms of the length of the alignment, but our results confirmed that the use of different alignment methods significantly affected tree topology. For instance, when using the alternative programs MUSCLE [20] and clustalw [21], only 7,22% of the trees had exactly the same topology. Moreover, we observed that the choice of the phylogenetic reconstruction method was also a source of variation. When comparing the trees produced using four alternative evolutionary models, we observed that only 9.9% of the trees presented the same topology in all models, and only 33% had two or more models pointing to the same topology. Thus, our results confirm previous findings [19] that topological variation may result from alignment uncertainty and extend this conclusion to the case of uncertainty in the specification of an evolutionary model. Besides alignment uncertainty and model misspecification, many other methodological aspects such as the modelling of co-variation or the assignment of proportion of invariable sites are subject to uncertainty and thus may also affect the levels of topological variation. That the choice of different parameters or methodologies introduces topological variations in phylogenies reconstructed from exactly the same sequences and that the levels of variation are similar to those observed when comparing trees from different genes, suggest that the lack of sufficient accuracy of current phylogenetic methods is likely to be an important source for the observed topological variation. This is especially true when the methods are used automatically without carefully selecting the parameters. Alternatively, one might argue that the small overlap between the topologies resulting from the use of different models/alignment methods results from the fact that only one of the methods is accurate and able to reconstruct the underlying true phylogeny. To further assess the accuracy of the phylogenetic methods used here under in a more controlled framework, we performed simulations of sequence evolution along the branches of the T60. For this we used as a seed 50 yeast sequences and simulated their evolution using the program ROSE [22]. Although in this case there is a true underlying phylogeny which is the same for all genes, in 70% of the cases, the phylogenetic reconstruction did not reconstruct the correct topology. A tree reconstructed from the concatenation of their alignments, however, was able to recover the original T60, topology.

### Conclusions

Altogether, our results show that, despite high levels of topological variations, the gene-concatenation approach can fairly recover the strongest phylogenetic signals present across single-gene phylogenies. As a result, most of the nodes in such a species tree do represent topologies that are widely represented across the genome. Our analysis only reflects the topological variation found in the yeast phylome and thus phylogenies of genes not present in *S. cerevisiae* are not taken into account. However, we consider that the circa 6000 phylogenies used do provide a broad enough sample to assess the strength of the topology of the species tree. The fact that we found no significant bias in terms of node support for the set of widespread genes (Figure 1C), suggest that other species phylomes are likely to provide similar results.

Despite the overall high support for most of the nodes in the species tree, some partitions of the species tree include topologies that are poorly represented in the phylome. Additionally, such conflicting nodes are more prone to variations when different taxonomic samples are used and are therefore less certain to be correct. Levels of topological support across a complete phylome provide a direct approach to identify such conflicting nodes. This measure is completely independent of bootstrap analyses, which only provide information on the support of the different partitions from the alignment in which the tree is based. Thus, as we have identified in our analyses, high bootstrap supports do not necessarily indicate highly represented topologies. As a way to identify conflicting nodes and to incorporate genome-wide information on species trees, we propose to map gene-tree variability (phylome support) levels on the nodes of the species tree. This information could be used to mark, or eventually collapse, low represented (<50%) nodes so that our uncertainty on certain areas of the tree of life is properly represented. Moreover, our approach could be used to compare alternative phylogenomic approaches in terms of their representativeness across large samples of single-gene phylogenies. A firm candidate for this comparison is the super-tree approach, which combines information from single copy genes that should not necessarily be widespread [23]. When used over fungal datasets, this approach has resulted in similar topologies to that produced by gene concatenation [8], [24], but the former were found to have less support in the literature [24].

The high levels of topological variations in single-gene phylogenies combined with the uncertainty on the inferred species trees may mislead further phylogenetic analyses such as the inference of orthology. In this respect we have shown that a relaxed interpretation may overcome the pitfalls of a strict reconciliation algorithm. In this direction, reconciliation algorithms that incorporate uncertainty in the gene and the species trees [25] or species-overlap algorithms [4], [26] may represent promising alternatives to standard phylogeny-based methods to predict orthology. Finally, our results suggest that a significant part of the topological variation among gene-trees may result from methodological uncertainty. In this study we have used molecular data from fungal genomes. The conclusions raised here are likely to be valid for other eukaryotic phyla. However, high levels of horizontal gene transfer across prokaryotic genomes, and perhaps certain unicellular eukaryotes, may invalidate the gene-concatenation approach as a means to infer a representative phylogeny. In such cases, besides the inherent levels of methodological noise discussed here, the topological variation in a genome will also reflect alternative evolutionary histories.

## Materials and Methods

### Sequence data

Proteins encoded in 60 fully-sequenced fungal genomes were downloaded from several databases (Supplementary table S1). Additionally, genomes from *Homo sapiens* and *Arabidopsis thaliana* were downloaded from ensembl (www.ensembl.org). The final database comprises 626,834 unique protein sequences.

### Yeast phylome reconstruction

We used the pipeline described in [12]. In contrast to family-based methods in which first sequences are clustered into groups based on pair-wise comparisons, for instance using MCL clustering, the phylome approach uses one genome as a seed to find putative homologs, just as a phylogeneticist would do to reconstruct the evolution of a protein of interest. This approach maximizes the coverage over the seed genome and being independent of the parameters of the clustering algorithm [4]. For each *Saccharomyces cerevisiae* “seed” protein a Smith-Waterman [27] search was used to retrieve, from the abovementioned database, a set of proteins with a significant similarity (E-val<10^−3^). Only sequences that aligned with a continuous region representing more than 33% of the query sequence were selected. These sequences are considered putative homologs and are aligned with MUSCLE 3.6 [20]. Positions in the alignment with gaps in more than 10% of the sequences were trimmed as described in [4]. Neighbour Joining trees were derived using scoredist distances as implemented in BioNJ [28]. PhyML aLRT version [29], [30] was used in to derive Maximum Likelihood (ML) trees. Four different evolutionary models were used for each seed sequence (JTT, WAG, Blosum62 and VT). In all cases, a discrete gamma-distribution model with four rate categories plus invariant positions was used, estimating the gamma parameter and the fraction of invariant positions from the data. The evolutionary model best fitting the data was determined by comparing the likelihood of the used models according to the AIC criterion [31]. The resulting 22,352 alignments and 111,760 phylogenetic trees for the four different generated phylomes can be publicly accessed in phylomeDB [12] (http://www.phylomedb.org).

### Reconstruction of the fungal trees of life

To reconstruct the T60 fungal species tree we proceeded as follows. Based on the orthology relationships derived from the yeast phylome (see below), we selected 69 proteins that were present in at least 58 of the 60 fungal organisms and show one-to-one orthology relationships in these species. The alignments of these proteins were concatenated into a single alignment, which was then trimmed to remove positions with gaps in more than 50% of the organisms. The resulting alignment comprises 31,123 amino acid positions. The tree was constructed using a Maximum Likelihood approach as implemented in the PhyML program [29], using a discrete gamma-distribution model with four rate categories plus invariant positions. The gamma parameter and the fraction of invariant positions were estimated from the data. The evolutionary model used for the analysis was WAG, as it was the model best fitting 61 of the 69 individual alignments.

The same procedure was also applied for the T21, T12a and T12b trees. Also using WAG as a model. In all cases the alignments were trimmed to eliminate columns with gaps in more than 50% of the positions. T21 is derived from a concatenation of 1137 protein families present in all 21 species. The final alignment included 28,3974 amino acid sites. T60 and T21 showed similar topologies with only the relative clustering of *Debaryomyces hansenii* and *Candida guillermondii* differing between the two trees. T12a included 2007 proteins present in all species and 580,514 positions. And, finally T12b comprised 217 widespread proteins and 95,528 positions. Support values were computed by bootstrap analysis of 100 replicates, unless indicated otherwise. The topologies in these two trees are fully compatible to that of T60.

### Simulations of sequence evolution

We used Rose [22] to generate simulated sequences from 50 yeast proteins that were chosen randomly among the ones used in the construction of T21. The simulations included insertions and deletions with a probability of 0.03. The other parameters for the simulation were the ones described in [32]. We also used the same strategy to infer the patterns of rate heterogeneity of the seed proteins. In short, we used TreePuzzle [33] assuming a 16 rate gamma distribution and for each position in the alignment we took the category and associated relative rate that contributed the most to the likelihood. These rates of heterogeneity were used by rose to model the evolution of the seed sequences along the T60 tree. The resulting simulated sequences were used to create a maximum likelihood tree using the WAG evolutionary model. Additionally, a species tree from the concatenated alignments was also reconstructed.

### Inference of duplication and speciation events and benchmark of orthology assignments

We used two alternative phylogeny-based methods to derive orthology relationships on the 60-species phylome. First, we used a previously described species-overlap algorithm [4] to map duplication and speciation events on the trees. In short, the algorithm starts at the seed protein used to generate the tree and runs through the internal nodes of the tree until it reaches the root. Trees were rooted at the midpoint. At each node, two daughter tree partitions are defined. If the two partitions share any species, the node is defined as a duplication node. Otherwise the node is defined as a speciation node. Once all the nodes have been classified, the algorithm establishes the orthologous and paralogous relationships between the seed protein and the rest of the proteins included in the tree.

Next, a strict tree-reconciliation algorithm was used [17]. In this case, every tree of the phylome is compared to the topology in the species tree by comparing the specific sets of species contained by all tree splits. The strict reconciliation algorithm maps the gene tree to the species tree and any incongruence is explained in terms of the minimal set of duplication and gene-loss events necessary to derive the observed gene tree topology from the one proposed in the species tree. These inferred duplication events are marked on the tree and orthology and paralogy relations are derived accordingly.

The orthology predictions derived from the phylome with the two strategies explained above, were compared to those made in the YGOB database [18]. We used this reference set to compute the number of true positives (TP), false positives (FP) and false negatives (FN) yielded by our method. For each method the sensitivity, S = TP/(TP+FN), and the positive predictive value, P = TP/(TP+FP) were computed.

### Topology scanning algorithm

The strategy used here to search for specific topologies within the phylome is based on an algorithm described earlier [34]. Perl scripts were written to implement this tree scanning algorithm to the specific scenarios considered here. In brief (see figure S6 in the supplementary material for more details), the algorithm proceeds sequentially throughout all internal edges of the tree, starting from each of the external nodes of the tree and proceed towards the root. Trees were rooted at the most distantly related species present in the tree, according to the topology in T60. At each internal node, two daughter partitions are generated and the species present in each such partition are tracked. The specific order in which the species appear in the tree can then be compared to specific scenarios. This algorithm was used to compare all the trees in a given phylome with the topology of the corresponding species tree. The algorithm considers only topological relationships among orthologous sequences. The phylome tree was considered to have a topology not compatible to that of the species tree if it contained a single species arrangement not found in the species tree and which could not be explained by gene loss events. Duplications found in the gene tree were always considered compatible and we only focused on the specific species arrangement within each partition resulting from the duplication. Proceeding in such way, we assure that only topological arrangements between orthologous genes are considered (duplications define paralogous relationships). Note that, since duplications may originate one-to-many or many-to-many orthology relationships it might be the case that the relationship with one co-ortholog is supporting the species tree topology while that with the other co-ortholog is rejecting it. Since we evaluate “full compatibility”, these trees were not considered compatible.

### Phylome support values

We define the “phylome support value” for a node as the percentage of trees in a phylome that present exactly the same topological arrangement of the partitions defined by its two daughter nodes. As indicated in figure 1-B, the two daughter nodes can define three (A,B,C) or four partitions (A,B,C,D) that might display three or fifteen alternative topologies, respectively. To compute the phylome support value we used the topology-scanning algorithm described above.

In this case, for any specific arrangement of three or four groups (see figure 1B) of species defined by a given node of the species trees we search for compatible partitions in all the trees in the phylome. Trees that did not have at least one species from each of the groups involved in the topology were not considered, because they do not provide information on that topology. That is, if the support for the topology ((A,B)C) is evaluated, we can only consider trees that contain at least one sequence from each of the three groups. Note that, in contrast to bootstrap supports that are only informative on the support for a single partition, the “phylome support value” takes into consideration the specific arrangement between several partitions and it is thus more informative.

## Supporting Information

Figure S1 (0.06 MB PDF)

Figure S2 (0.07 MB PDF)

Figure S3 (0.06 MB PDF)

Figure S4 (0.07 MB PDF)

Figure S5 (0.32 MB PDF)

Figure S6 (0.12 MB PDF)

Table S1 (0.08 MB PDF)

Table S2 (0.12 MB PDF)

Table S3 (0.19 MB PDF)

Table S4 (0.14 MB PDF)
