# Supplementary material for: The Tree versus the Forest: The Fungal Tree of Life and the Topological Diversity within the Yeast Phylome
Source: PLoS One. 2009 Feb 3;4(2):e4357. doi: 10.1371/journal.pone.0004357 (PMC2629814; doi:10.1371/journal.pone.0004357)
Supplement: Figure S1 — (0.06 MB PDF) [file pone.0004357.s001.pdf]

Figure S1 T21

Phylogenetic tree representing the evolutionary relationships among 21 fungal species from the Saccharomycotina group. The tree was build using ML analysis on a concatenated alignment of 1,137 widespread proteins. Numbers indicate phylome and bootstrap supports, as in figure 1. In this case only 10 bootstrap samples were ran, due to the high computational demands.

S1

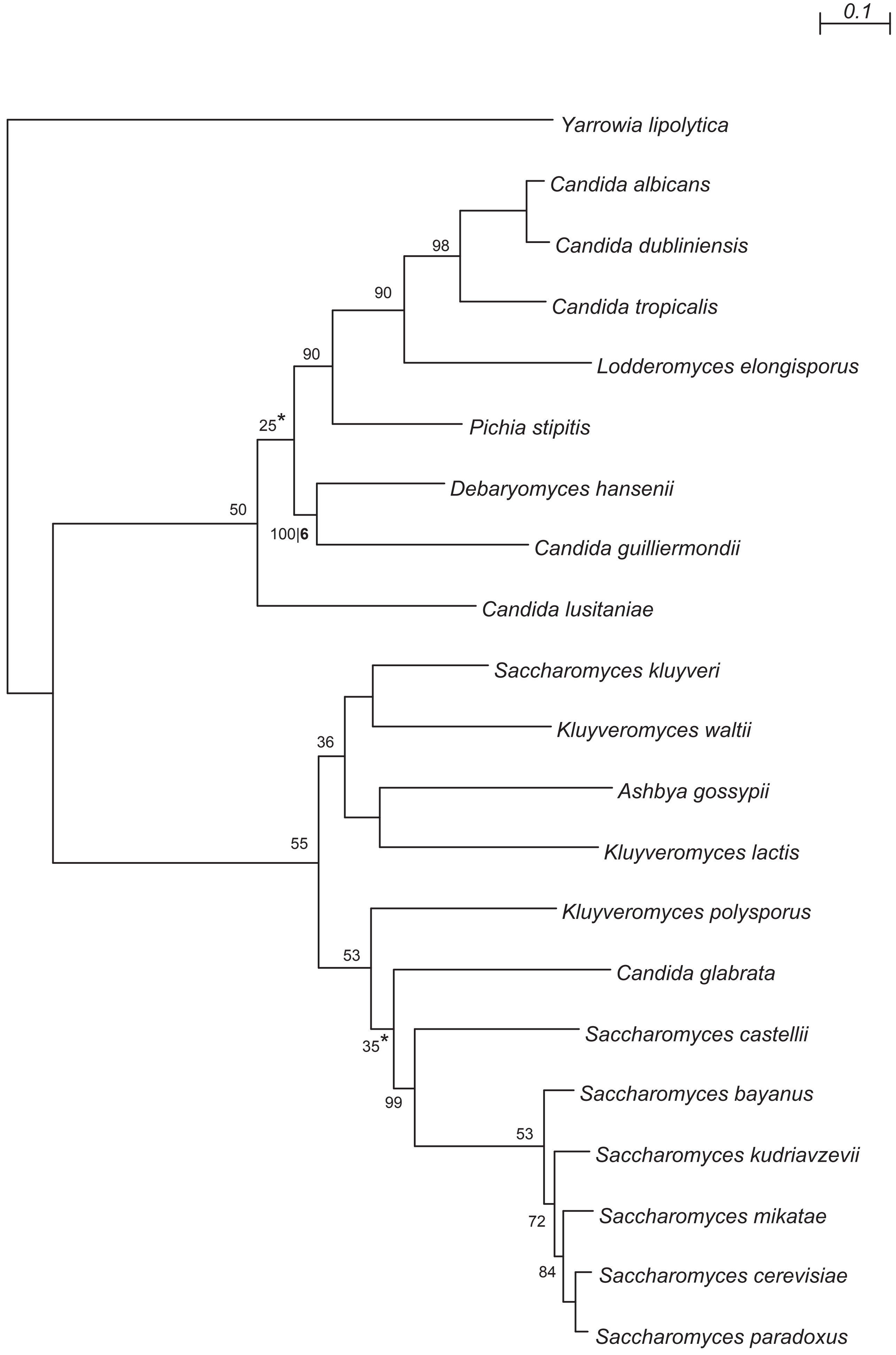

0.1
Yarrowia lipolytica
Candida albicans
Candida dubliniensis
Candida tropicalis
Lodderomyces elongisporus
Pichia stipitis
Debaryomyces hansenii
Candida guilliermondii
Candida lusitaniae
Saccharomyces kluyveri
Kluyveromyces waltii
Ashbya gossypii
Kluyveromyces lactis
Kluyveromyces polysporus
Candida glabrata
Saccharomyces castellii
Saccharomyces bayanus
Saccharomyces kudriavzevii
Saccharomyces mikatae
Saccharomyces cerevisiae
Saccharomyces paradoxus
98
90
90
25*
50
100|6
36
55
53
35*
99
53
72
84
