# Supplementary material for: The Tree versus the Forest: The Fungal Tree of Life and the Topological Diversity within the Yeast Phylome
Source: PLoS One. 2009 Feb 3;4(2):e4357. doi: 10.1371/journal.pone.0004357 (PMC2629814; doi:10.1371/journal.pone.0004357)
Supplement: Figure S2 — (0.07 MB PDF) [file pone.0004357.s002.pdf]

Figure S2 T12a

Phylogenetic tree representing the evolutionary relationships among 12 fungal species from the *Saccharomyces genus*. The tree was build using ML analysis on a concatenated alignment of 2,007 widespread proteins. Numbers indicate phylome and bootstrap supports, as in figure 1.

S2

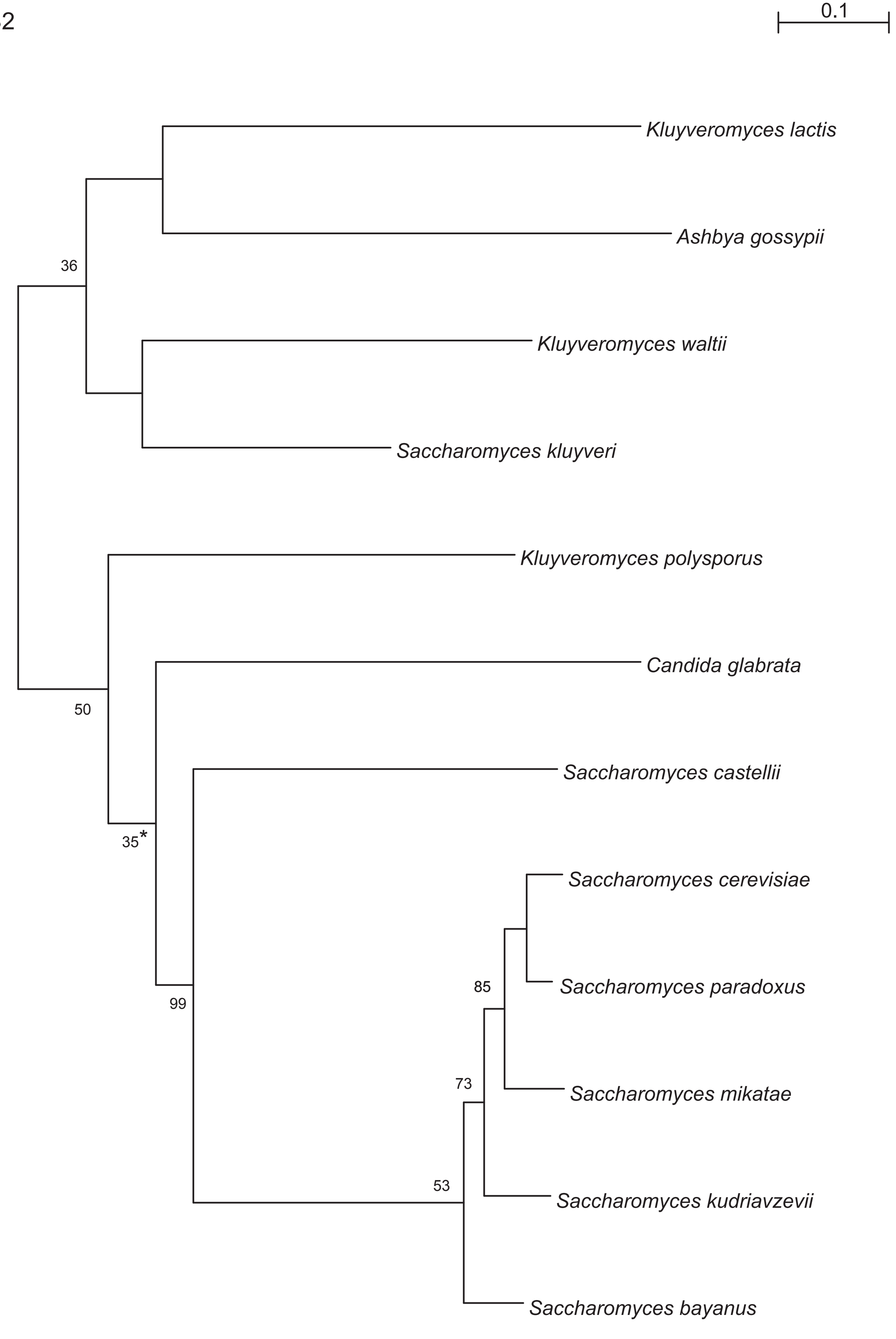
0.1
Kluyveromyces lactis
Ashbya gossypii
36
Kluyveromyces waltii
Saccharomyces kluyveri
Kluyveromyces polysporus
50
Candida glabrata
Saccharomyces castellii
35*
Saccharomyces cerevisiae
85
Saccharomyces paradoxus
99
Saccharomyces mikatae
73
53
Saccharomyces kudriavzevii
Saccharomyces bayanus
