# Supplementary material for: The Tree versus the Forest: The Fungal Tree of Life and the Topological Diversity within the Yeast Phylome
Source: PLoS One. 2009 Feb 3;4(2):e4357. doi: 10.1371/journal.pone.0004357 (PMC2629814; doi:10.1371/journal.pone.0004357)
Supplement: Figure S3 — (0.06 MB PDF) [file pone.0004357.s003.pdf]

Figure S3 T12b

Phylogenetic tree representing the evolutionary relationships among 12 fungal species, sampled from the fungal groups. The tree was build using ML analysis on a concatenated alignment of 217 widespread proteins. Numbers indicate phylome and bootstrap supports, as in figure 1.

S3

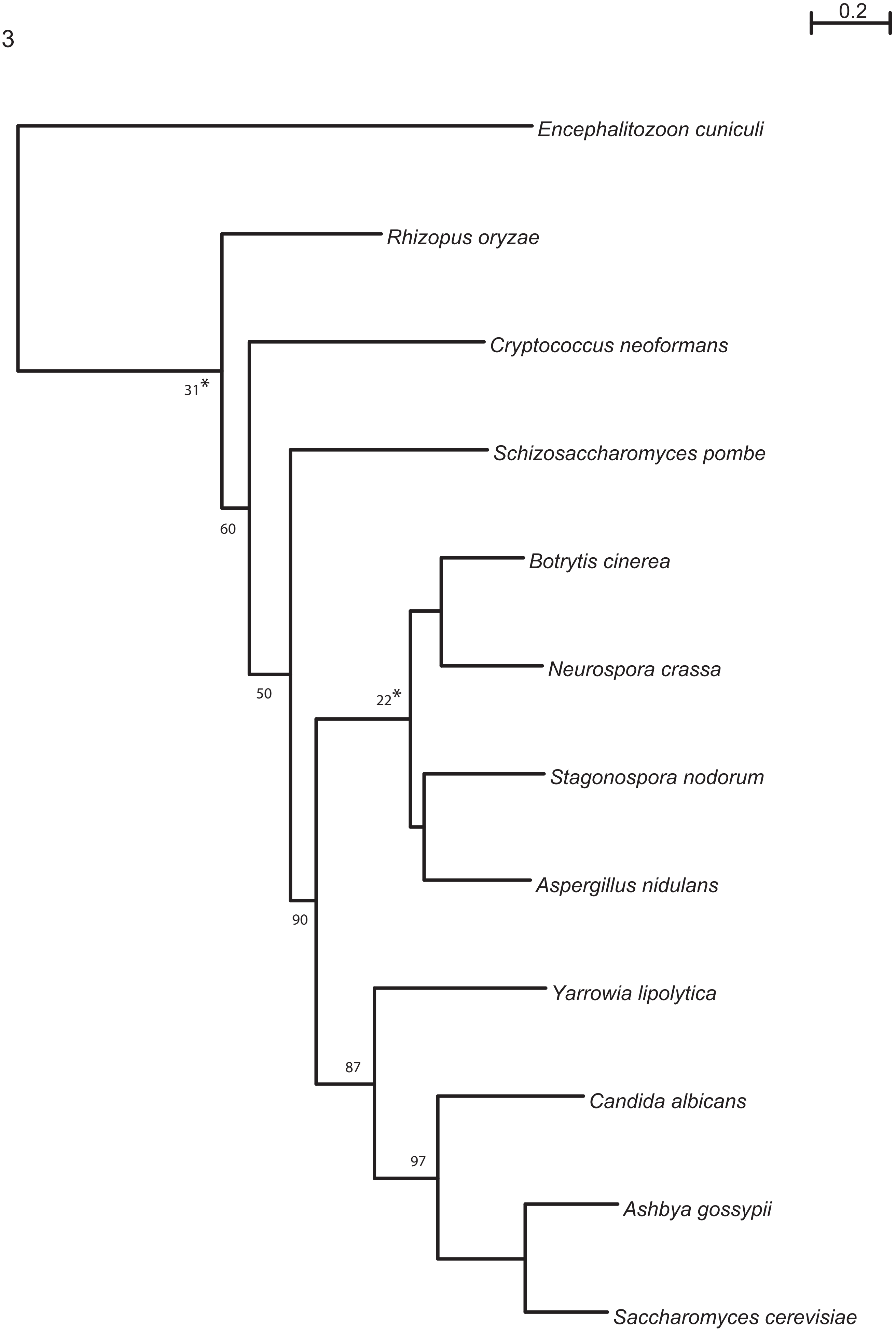
0.2
Encephalitozoon cuniculi
Rhizopus oryzae
Cryptococcus neoformans
Schizosaccharomyces pombe
Botrytis cinerea
Neurospora crassa
Stagonospora nodorum
Aspergillus nidulans
Yarrowia lipolytica
Candida albicans
Ashbya gossypii
Saccharomyces cerevisiae
31*
60
50
22*
90
87
97
