# Supplementary material for: The Tree versus the Forest: The Fungal Tree of Life and the Topological Diversity within the Yeast Phylome
Source: PLoS One. 2009 Feb 3;4(2):e4357. doi: 10.1371/journal.pone.0004357 (PMC2629814; doi:10.1371/journal.pone.0004357)
Supplement: Figure S4 — (0.07 MB PDF) [file pone.0004357.s004.pdf]

Figure S4 Branch support and phylome vs ST topological congruence

Percentage of trees in the phylome that are fully compatible with the topology of T60 (Y axis), at different statistical support thresholds in the nodes considered (X axis). The statistical support used is the minimum value of the approximate likelihood as computed by the Chi2-based parametric methodology or the non-parametric branch support based on a Shimodaira-Hasenawa-like procedure (option -3 in PhyML aLRT). These methodologies are described in [1]. The nodes with a statistical support lower than the given threshold were collapsed. We only considered trees in which less than 50% of the nodes were collapsed.

1. Anisimova M, Gascuel O (2006) Approximate likelihood-ratio test for branches: A fast, accurate, and powerful alternative. Syst Biol 55: 539-552.

S4.-

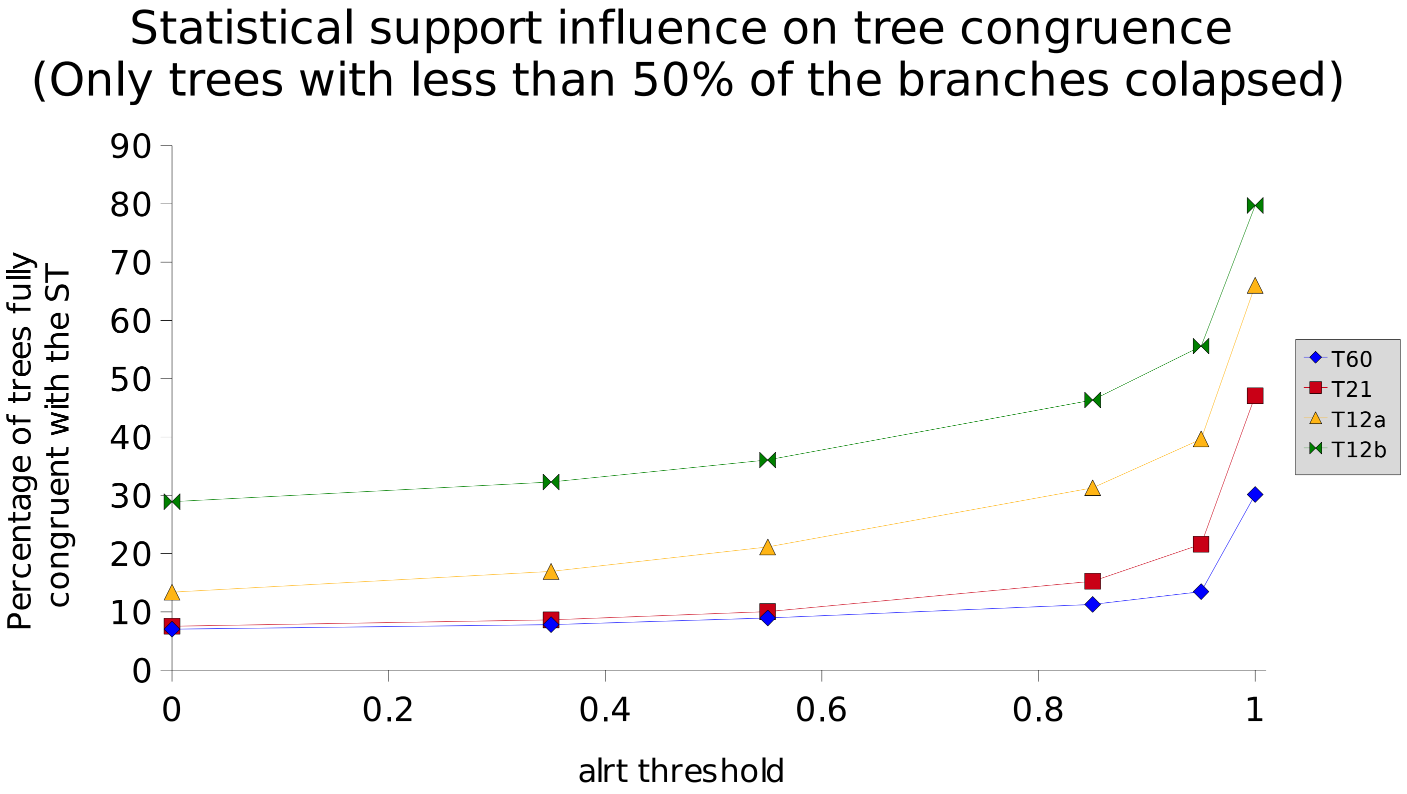
Statistical support influence on tree congruence
(Only trees with less than 50% of the branches colapsed)
Percentage of trees fully congruent with the ST
alrt threshold
0
10
20
30
40
50
60
70
80
90
0
0.2
0.4
0.6
0.8
1
T60
T21
T12a
T12b
