# Supplementary figures and images for: The Tree versus the Forest: The Fungal Tree of Life and the Topological Diversity within the Yeast Phylome
Source: PLoS One. 2009 Feb 3;4(2):e4357. doi: 10.1371/journal.pone.0004357 (PMC2629814; doi:10.1371/journal.pone.0004357)

**Figure S5**

Species trees based on randomly chosen sets of 50 (a,b,c), 40 (a,b,c) and 30 (a,b,c) species.

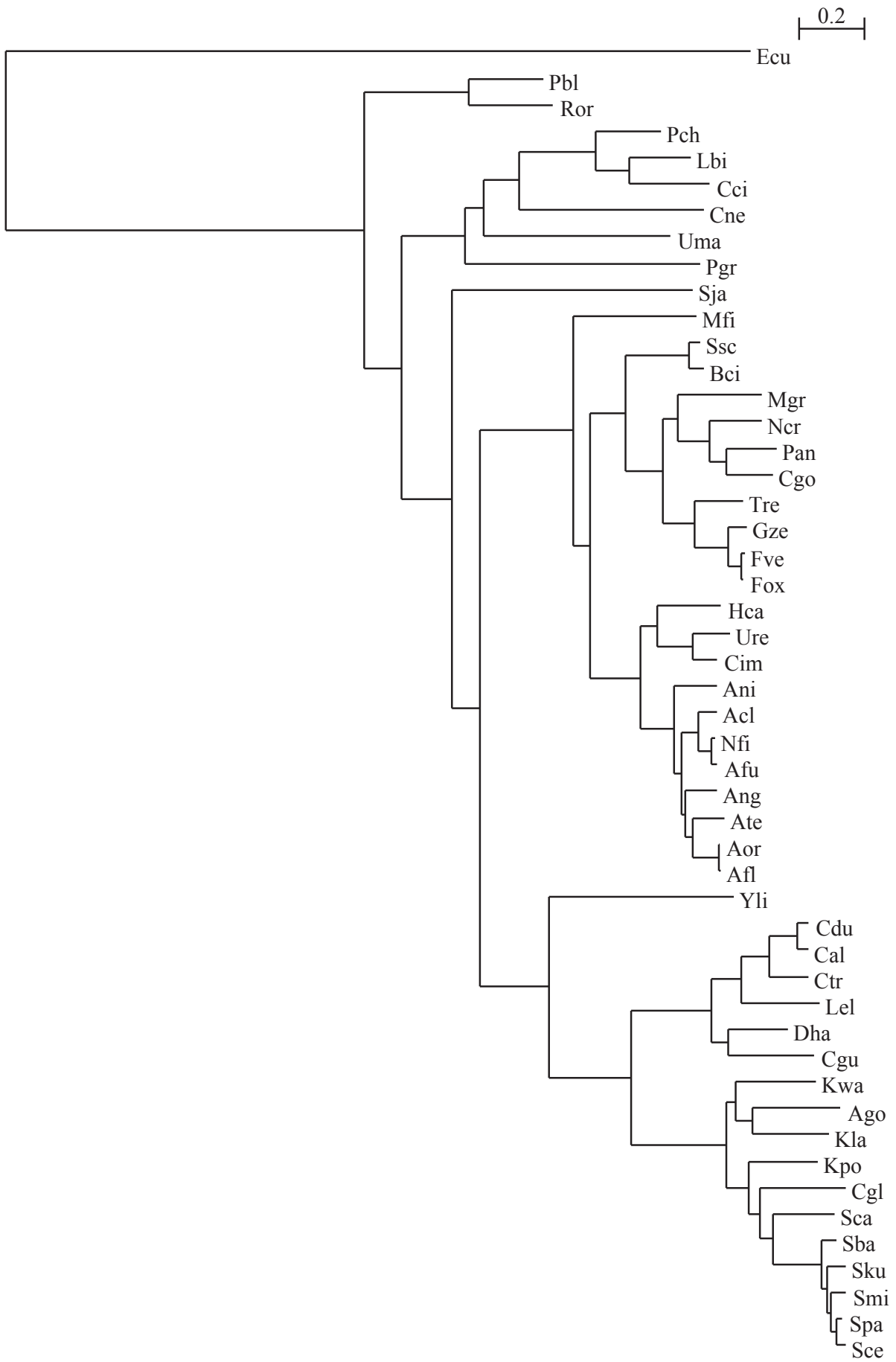

0.2

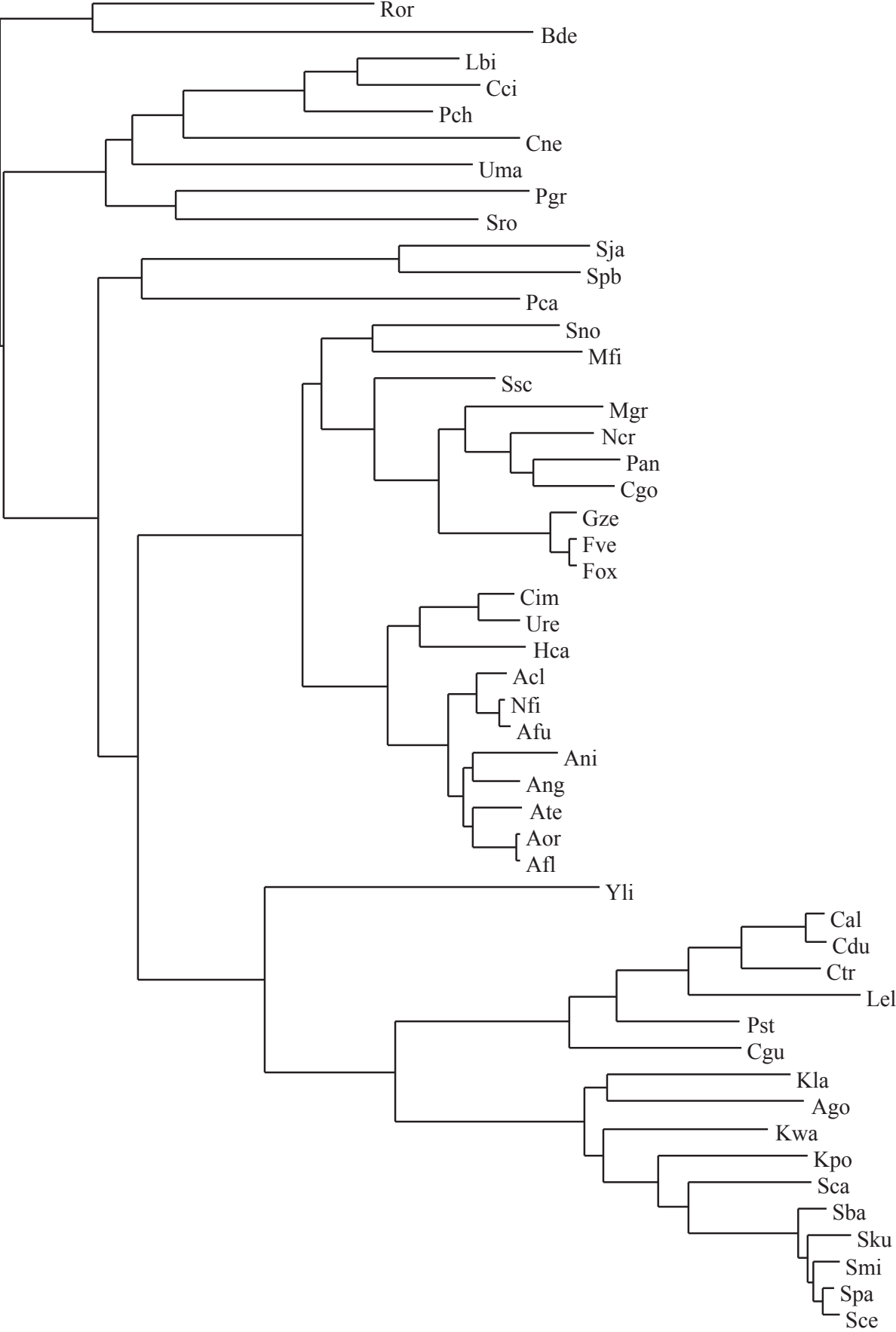

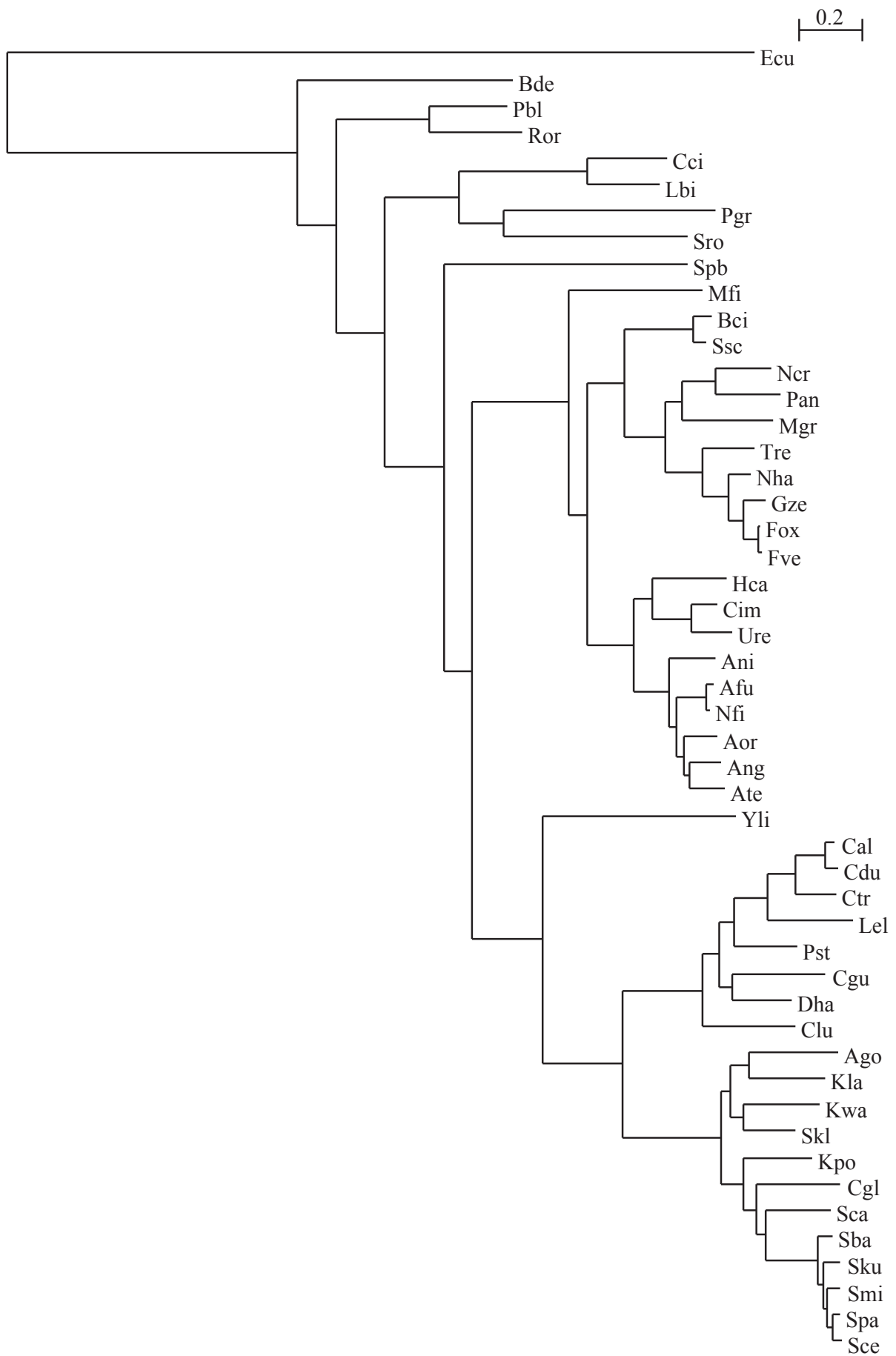

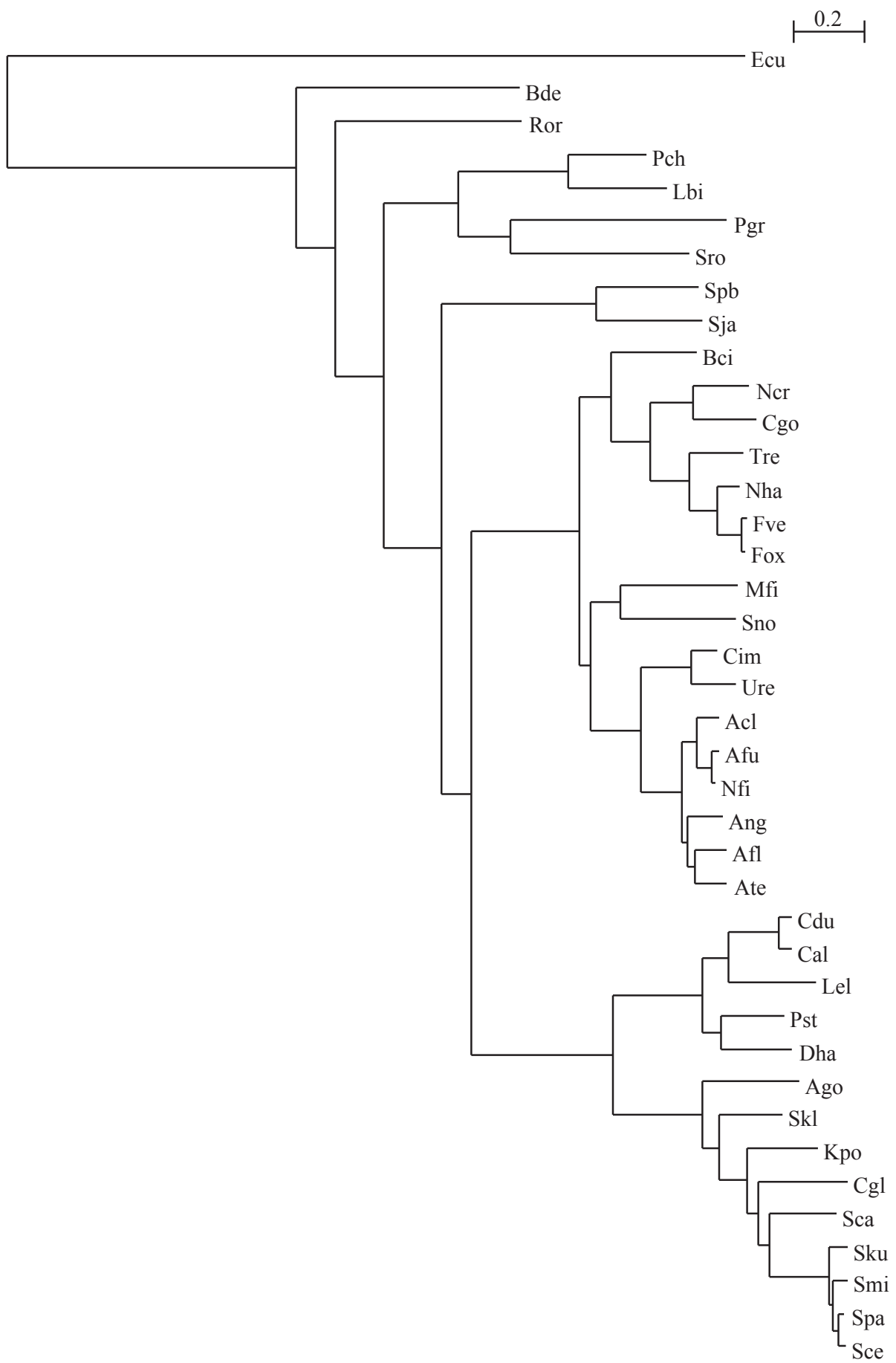

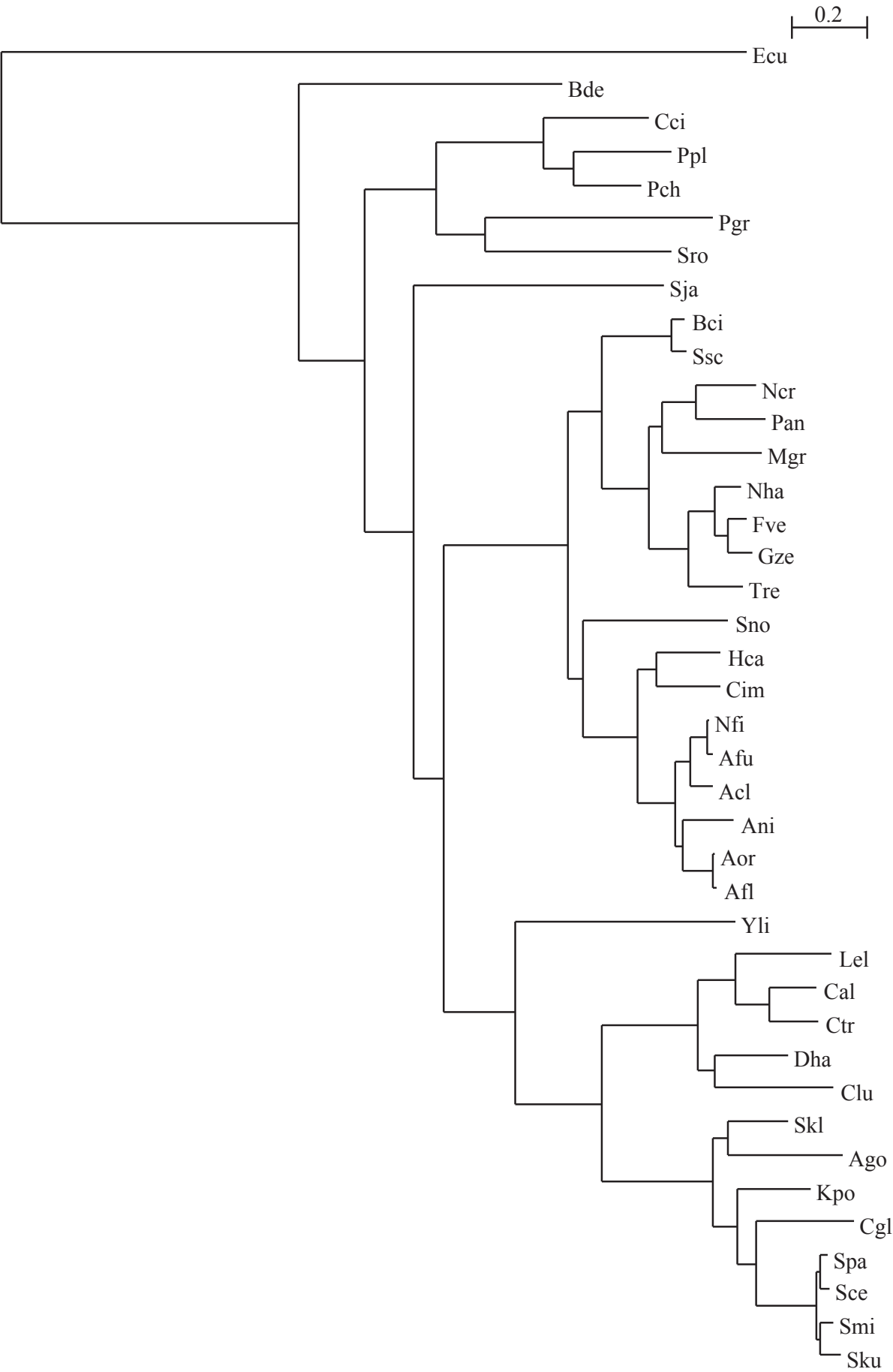

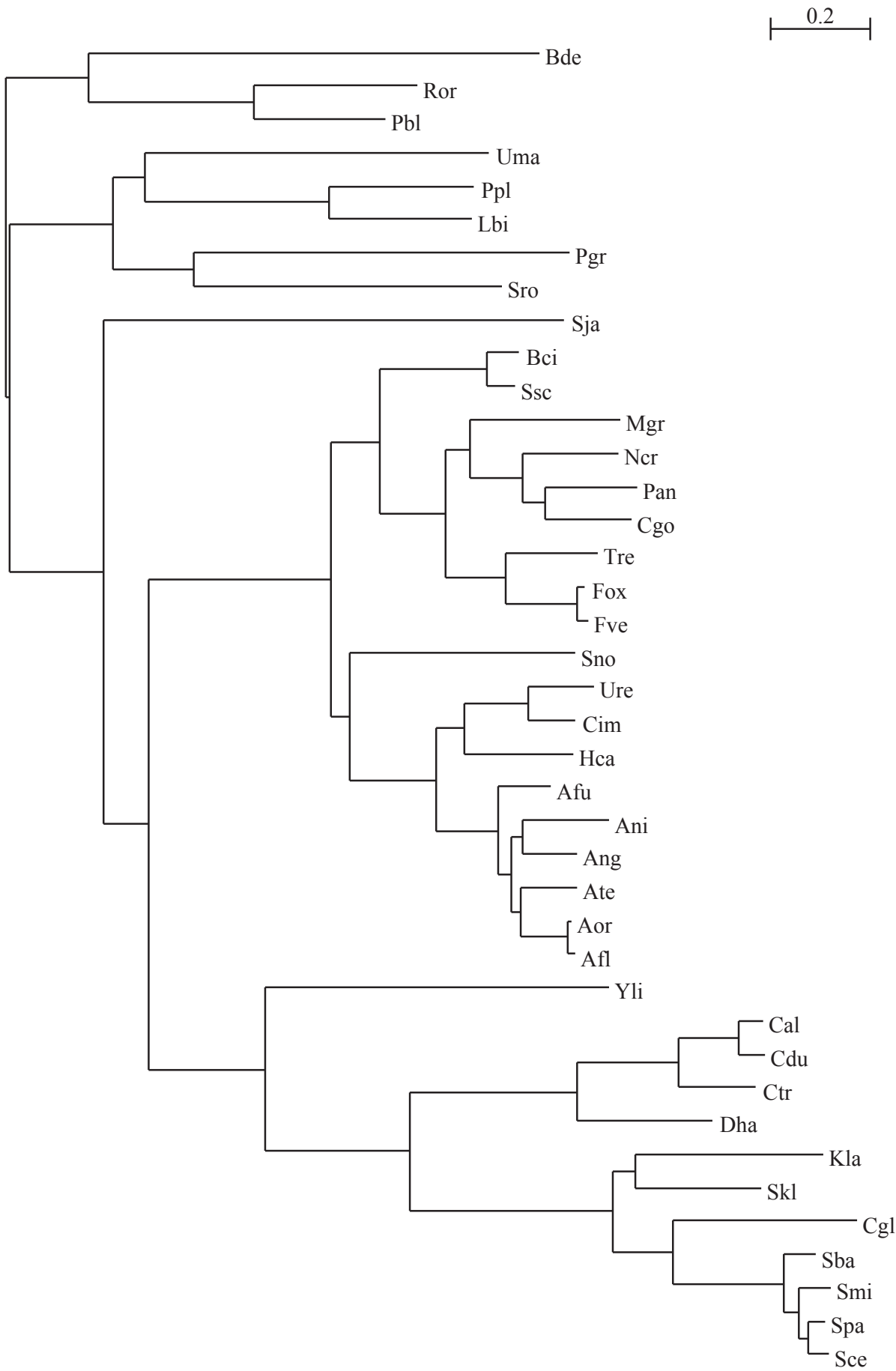

0.2

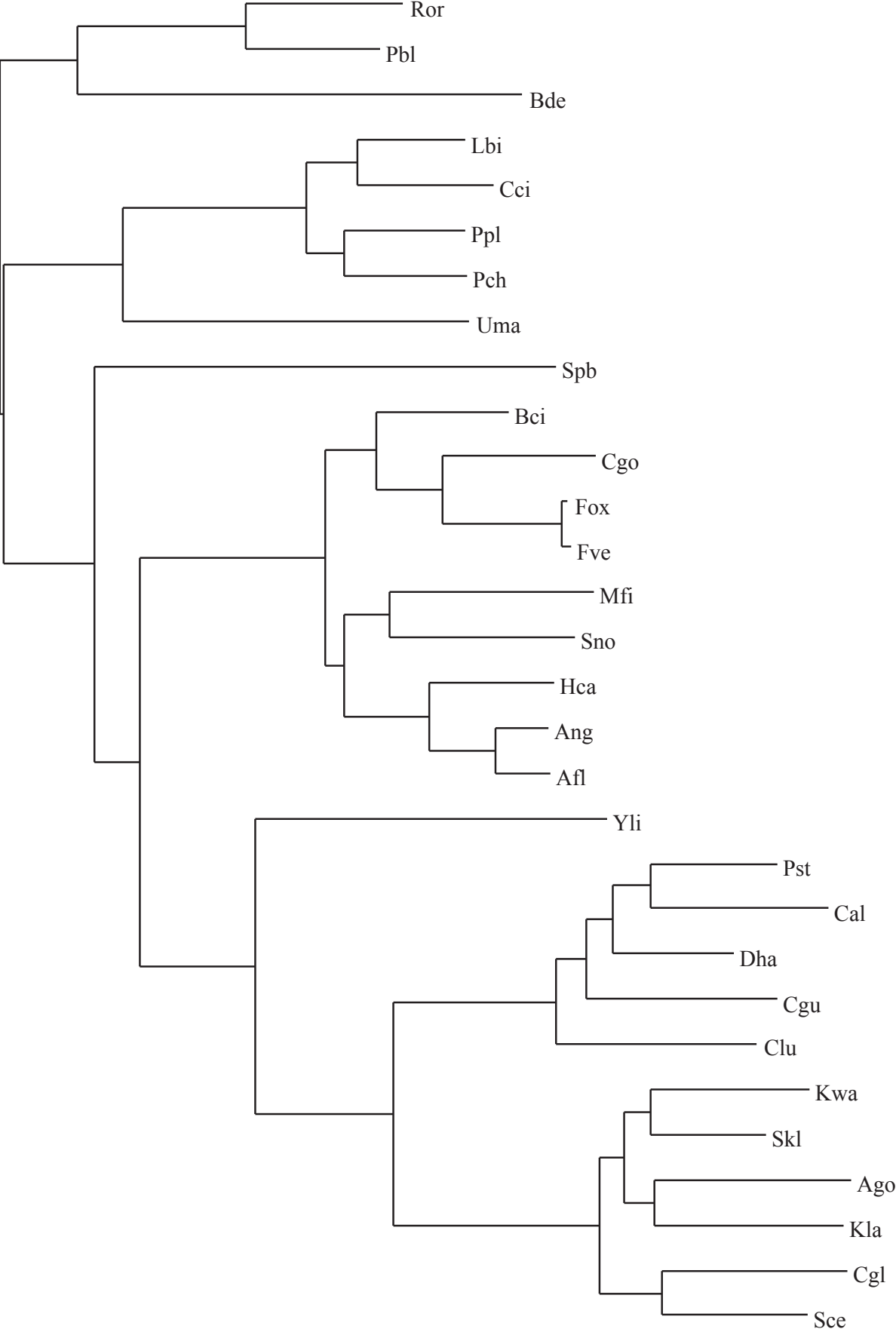

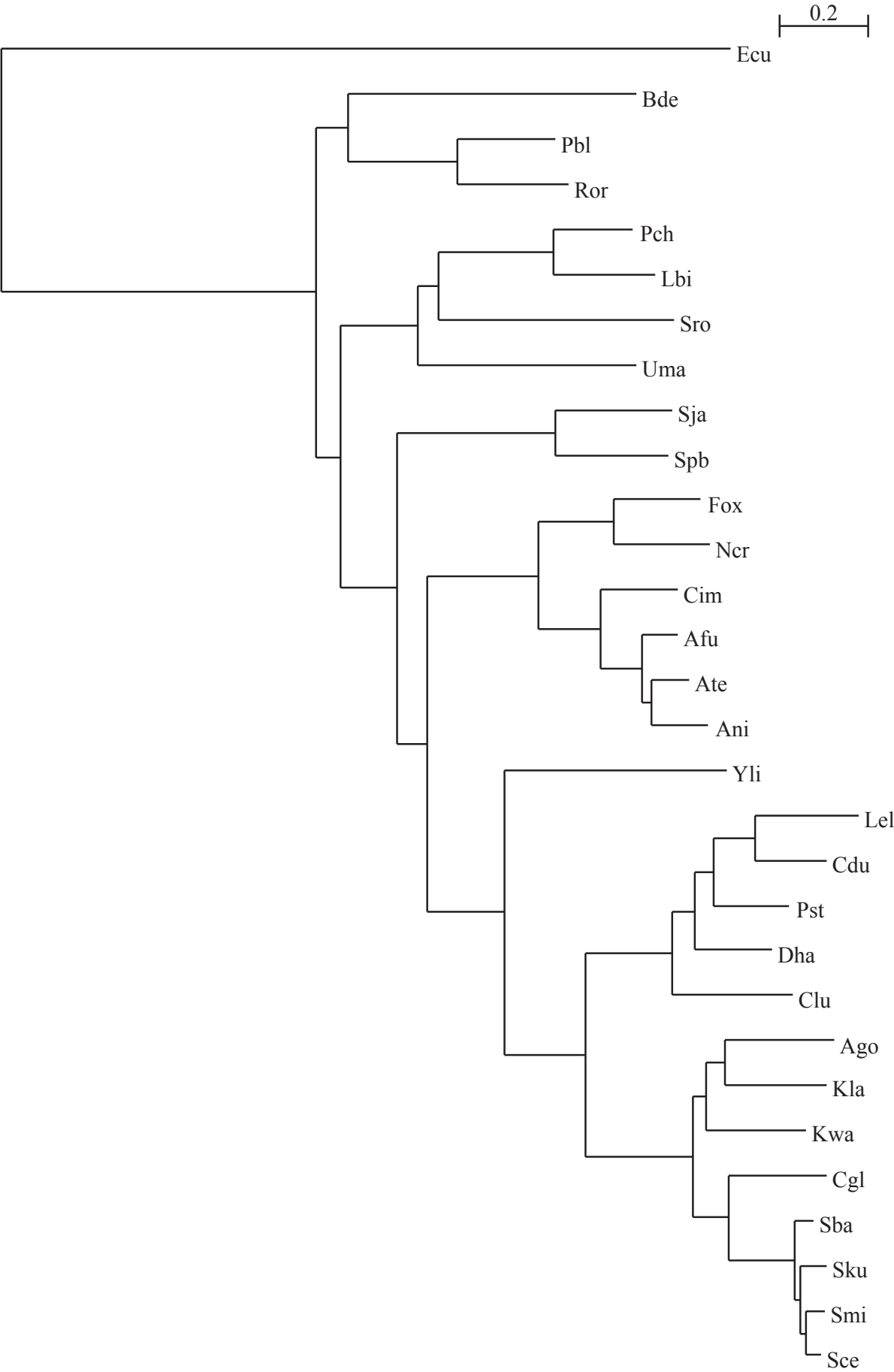

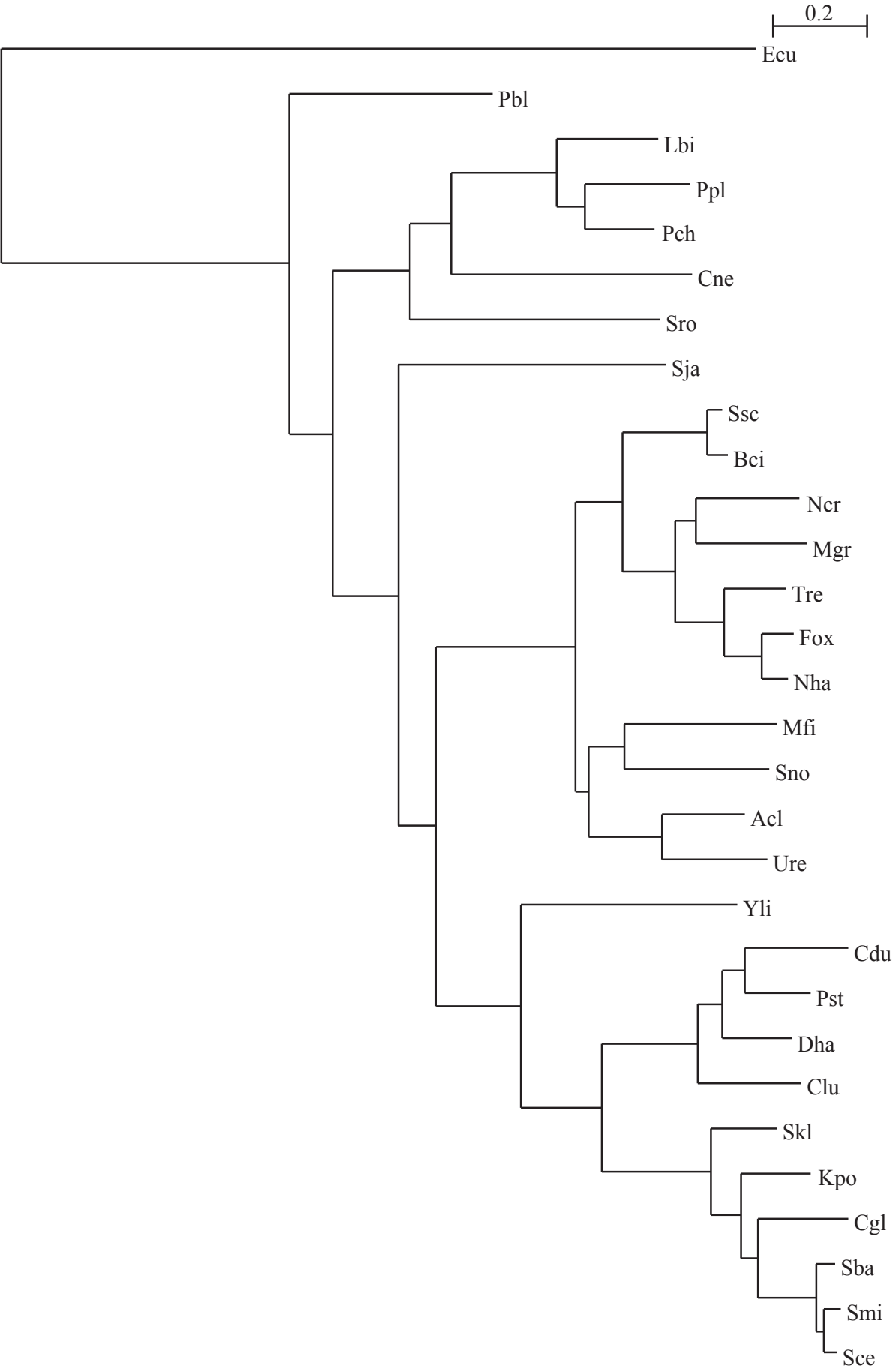

Supplement: Figure S5 — (0.32 MB PDF) [file pone.0004357.s005.pdf]
