# Supplementary material for: The Tree versus the Forest: The Fungal Tree of Life and the Topological Diversity within the Yeast Phylome
Source: PLoS One. 2009 Feb 3;4(2):e4357. doi: 10.1371/journal.pone.0004357 (PMC2629814; doi:10.1371/journal.pone.0004357)
Supplement: Figure S6 — (0.12 MB PDF) [file pone.0004357.s006.pdf]

Figure S6

Explanation on how the topology scanning algorithm works in different situations. Figure S6a shows a tree that is congruent with the species tree despite the loss of two species. Figure S6b shows a tree that is not congruent with the species tree due to the rearrangement of two of its branches. Since no species overlap exists we do not consider this node as a duplication node as there is no evidence for such an assumption. Figure S6c represents a congruent tree with a duplication node as predicted by the species overlap algorithm.

S6.-

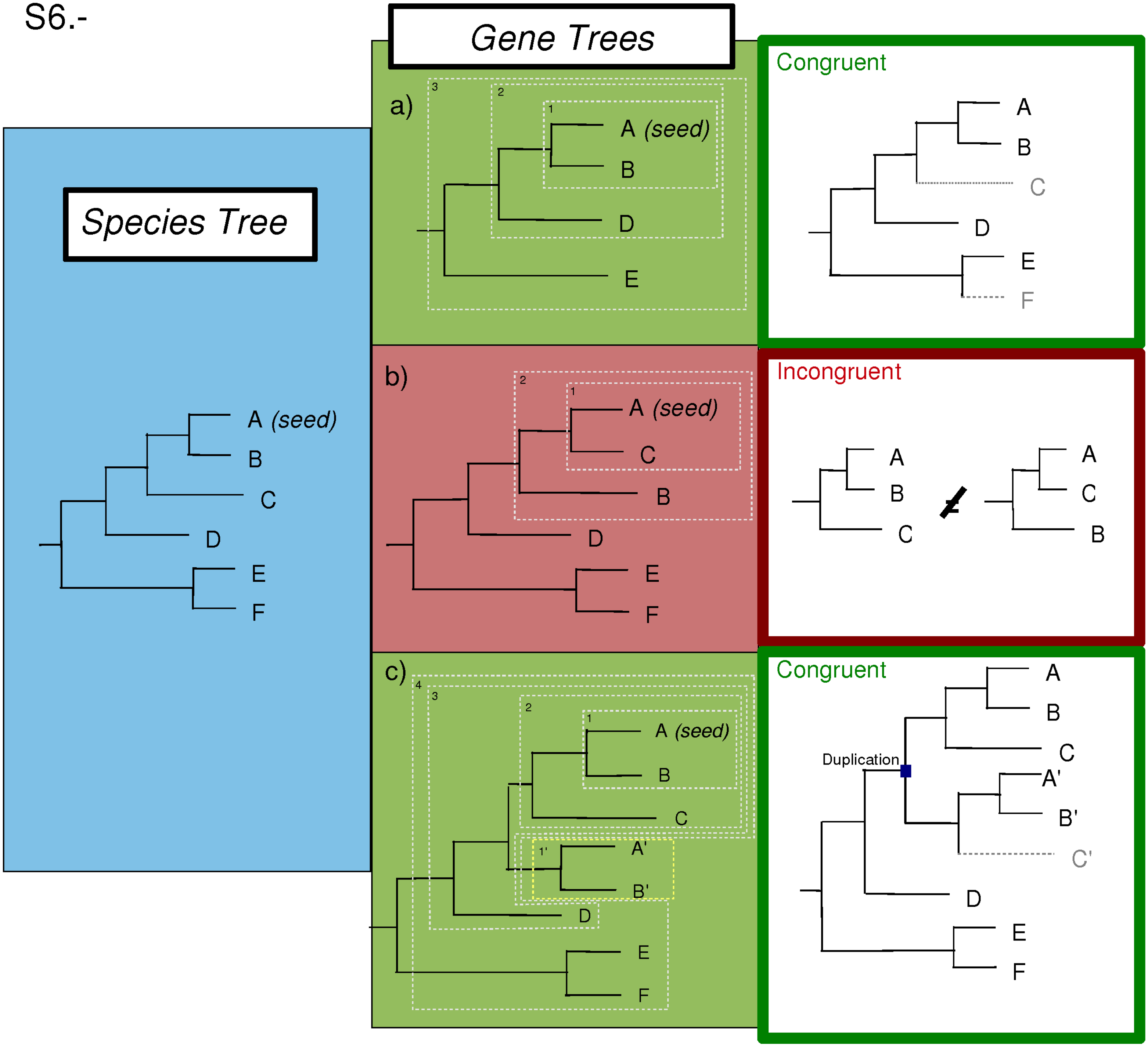

Gene Trees
Species Tree
A (seed)
B
C
D
E
F
a)
b)
c)
Congruent
Incongruent
Duplication
A'
B'
C'
