# Supplementary material for: The Tree versus the Forest: The Fungal Tree of Life and the Topological Diversity within the Yeast Phylome
Source: PLoS One. 2009 Feb 3;4(2):e4357. doi: 10.1371/journal.pone.0004357 (PMC2629814; doi:10.1371/journal.pone.0004357)
Supplement: Table S1 — (0.08 MB PDF) [file pone.0004357.s007.pdf]

Table S1

Species included in the 60-species phylomes and their genomic coverage. For each species, “proteins included” column indicates the number of proteins present in trees of the yeast phylome and the percentage they represent; “trees” column indicates the number of trees in the phylome with proteins from that species (and the percentage from the phylome it represents). “Source” indicates the database from which the protein data for that species was retrieved. The last four columns indicate if this species was considered in each of the indicated species trees (shadowed boxes). Sources included are: JGI (http://www.jgi.doe.gov), Broad Institute (http://www.broad.mit.edu), YGOB (http://wolfe.gen.tcd.ie/ygob/), SGD (www.yeastgenome.org), Fungal genomes (http://fungalgenomes.org), Genolevures (http://cbi.labri.fr/genolevures/), integr8 (http://www.ebi.ac.uk/integr8), candida genome database (http://www.candidagenome.org), NCBI (http://www.ncbi.nlm.nih.gov).

Supplementary table 1

	Code	Organism name	Source	Proteins	Trees	T60	T21	T12a	T12b
Saccharomyces sensu stricto	Sce	Saccharomyces cerevisiae	SGD	5811 (86%)	5804 (100%)	■	■	■	■
	Spa	Saccharomyces paradoxus	Fungal Genomes	5356 (95%)	5396 (93%)	■	■	■	
	Smi	Saccharomyces mikatae	Fungal Genomes	5136 (90%)	5263 (91%)	■	■	■	
	Sku	Saccharomyces kudriavzevii	Fungal Genomes	5027 (83%)	5114 (88%)	■	■	■	
	Sba	Saccharomyces bayanus	SGD	5382 (91%)	5464 (94%)	■	■	■	
Saccharomyces complex	Sca	Saccharomyces castellii	YGOB	5253 (92%)	5165 (89%)	■	■	■	
	Cgl	Candida glabrata	Genolevures	4866 (93%)	5032 (87%)	■	■	■	
	Kpo	Kluyveromyces polysporus	YGOB	5004 (91%)	5129 (88%)	■	■	■	
	Ago	Ashbya gossypii	NCBI	4314 (91%)	4815 (83%)	■	■	■	■
	Kla	Kluyveromyces lactis	NCBI	4542 (85%)	5030 (87%)	■	■	■	
	Kwa	Kluyveromyces waltii	YGOB	4482 (85%)	4986 (86%)	■	■	■	
	Skl	Saccharomyces kluyveri	Fungal Genomes	4267 (74%)	4736 (82%)	■	■	■	
Candida cluster	Cal	Candida albicans	Candida Genome Database	4063 (66%)	4291 (74%)	■	■	■	■
	Cdu	Candida dubliniensis	Fungal Genomes	3952 (59%)	4165 (72%)	■	■		
	Ctr	Candida tropicalis	Broad Institute	4089 (65%)	4185 (72%)	■	■		
	Lel	Lodderomyces elongisporus	Broad Institute	3788 (65%)	4082 (70%)	■	■		
	Pst	Pichia stipitis	JGI	4162 (71%)	4277 (74%)	■	■		
	Dha	Debaryomyces hansenii	Integr8	4132 (65%)	4323 (74%)	■	■		
	Cgu	Candida guilliermondii	Broad Institute	4045 (68%)	4202 (72%)	■	■		
	Clu	Candida lusitaniae	Broad Institute	3831 (64%)	4180 (72%)	■	■		
Dipodascaceae	Yli	Yarrowia lipolytica	Integr8	3882 (59%)	4009 (69%)	■	■		■
Leotiomycetes	Bci	Botrytis cinerea	Broad Institute	3503 (21%)	3625 (62%)	■			■
	Ssc	Sclerotinia sclerotiorum	Broad Institute	3554 (24%)	3762 (65%)	■			
Sodariomycetes	Mgr	Magnaporthe grisea	Broad Institute	3566 (27%)	3680 (63%)	■			
	Ncr	Neurospora crassa	Broad Institute	3392 (31%)	3708 (64%)	■			■
	Cgo	Chaetomium globosum	Broad Institute	3207 (28%)	3458 (60%)	■			
	Pan	Podospora anserina	Fungal Genomes	3525 (27%)	3637 (63%)	■			
	Tre	Trichoderma reesei	JGI	3663 (40%)	3799 (65%)	■			
	Gze	Fusarium graminearum	Integr8	3905 (33%)	3755 (65%)	■			
	Fox	Fusarium oxysporum	Broad Institute	4375 (24%)	3780 (65%)	■			
	Fve	Fusarium verticillioides	Broad Institute	3965 (27%)	3736 (64%)	■			
	Nha	Nectria haematococca	JGI	4671 (29%)	3802 (66%)	■			
Dothideomycetes	Sno	Stagonospora nodorum	Broad Institute	3883 (23%)	3783 (65%)	■			■
	Mfi	Mycosphaerella fijiensis	JGI	3472 (33%)	3606 (62%)	■			
Eurotiomycetes	Hca	Histoplasma capsulatum	Broad Institute	2993 (32%)	3415 (59%)	■			
	Cim	Coccidioides immitis	Broad Institute	3361 (32%)	3680 (63%)	■			
	Ure	Uncinocarpus reesii	Broad Institute	3159 (40%)	3491 (60%)	■			
	Acl	Aspergillus clavatus	TIGR	3873 (42%)	3855 (66%)	■			
	Afu	Aspergillus fumigatus	Broad Institute	3907 (40%)	3837 (66%)	■			
	Nfi	Neosartorya fischeri	TIGR	4063 (39%)	3845 (66%)	■			
	Ani	Aspergillus nidulans	NCBI	3629 (38%)	3729 (64%)	■			■
	Ang	Aspergillus niger	Broad Institute	4157 (29%)	3809 (66%)	■			
	Aor	Aspergillus oryzae	Integr8	4090 (33%)	3653 (63%)	■			
	Afl	Aspergillus flavus	Broad Institute	4144 (32%)	3770 (65%)	■			
	Ate	Aspergillus terreus	Broad Institute	3865 (37%)	3710 (64%)	■			
Taphrinomycotina	Spb	Schizosaccharomyces pombe	Integr8	3212 (64%)	3442 (59%)	■			■
	Sja	Schizosaccharomyces japonicus	Broad Institute	3076 (59%)	3287 (57%)	■			
	Pca	Pneumocystis carinii	Fungal Genomes	814 (20%)	965 (17%)	■			
Basidiomycota	Cci	Coprinus cinereus	Broad Institute	3210 (23%)	3310 (57%)	■			
	Cne	Cryptococcus neoformans	Integr8	3130 (47%)	3366 (58%)	■			■
	Lbi	Laccaria bicolor	JGI	3779 (18%)	3350 (58%)	■			
	Ppl	Postia placenta	JGI	4952 (28%)	3291 (57%)	■			
	Pch	Phanerochaete chrysosporium	JGI	3241 (32%)	3216 (55%)	■			
	Pgr	Puccinia graminis	Broad Institute	2966 (14%)	2956 (51%)	■			
	Sro	Sporobolomyces roseus	JGI	2713 (49%)	3024 (52%)	■			
	Uma	Ustilago maydis	Broad Institute	2858 (43%)	3287 (57%)	■			
Zygomycota	Ror	Rhizopus oryzae	Broad Institute	4782 (27%)	3193 (55%)	■			■
	Pbl	Phycomyces blakesleeanus	JGI	4482 (30%)	3386 (58%)	■			
Chitridiomycota	Bde	Batrachochytrium dendrobatidis	Broad Institute	2774 (31%)	2842 (49%)	■			
Microsporidia	Ecu	Encephalitozoon cuniculi	Integr8	698 (36%)	920 (16%)	■			■
