# Supplementary material for: The Tree versus the Forest: The Fungal Tree of Life and the Topological Diversity within the Yeast Phylome
Source: PLoS One. 2009 Feb 3;4(2):e4357. doi: 10.1371/journal.pone.0004357 (PMC2629814; doi:10.1371/journal.pone.0004357)
Supplement: Table S2 — (0.12 MB PDF) [file pone.0004357.s008.pdf]

Table S2

Level of phylome support for each possible topological arrangement of the nodes represented in the different species trees. For each node, the species represented by each of the three or four groups considered are indicated. Then, all possible topological arrangements of these groups (three possibilities for three groups and 15 for four groups) and the percentage of trees in the phylome that support that topology are indicated. The specific topology obtained in the species tree is shadowed and the most supported topology in the phylome is highlighted in red.

Supplementary table 2
(1 of 6)

T60 phylome:

Topologies 3	((A,B),C)	((A,C),B)	((B,C),A)
A=Cal B=Cdu C=Ctr	97,76	0,95	1,29
A=Cal-Cdu B=Ctr C=Lel	90,9	4,68	4,42
A=Cal-Cdu-Ctr B=Lel c=Pst	91	5,16	3,84
A=Cal-Cdu-Ctr-Lel B=Pst C=Dha	60,31	6,89	32,8
A=Cal-Cdu-Ctr-Lel-Pst B=Dha C=Cgu	37,31	13,68	49,01
A=Cal-Cdu-Ctr-Lel-Pst-Dha B=Cgu C=Clu	44,38	25,54	30,07
A=Fox B=Fve C=Gze	93,33	3,22	3,45
A=Fox-Fve B=Gze C=Nha	88,34	5,91	5,75
A=Fox-Fve-Gze B=Nha C=Tre	94,92	1,41	3,67
A=Lbi-Cci B=Pch-Ppl C=Cne	97,41	1,53	1,06
A=Lbi-Cci-Pch-Ppl B=Cne C=Uma	64,36	14,23	21,41
A=Afu B=Nfi C=Acl	96,72	1,5	1,78
A=Ure B=Cim C=Hca	97,94	1,3	0,75
A=Cgo B=Pan C=Ncr	66,92	15,39	17,7

Topologies 3	((A,B),C)	((A,C),B)	((B,C),A)
A=Cgo-Pan B=Ncr C=Mgr	84,5	7,91	7,59
A=Ror B=Pbl C=Bde	97,79	1	1,2
A=Spb B=Sja C=Pca	97,97	0,74	1,29
A=Aor B=Afl C=Ate	99,25	0,45	0,3
A=Aor-Afl B=Ate C=Ang	47,94	27,33	24,73
A=Aor-Afl-Ate B=Ang C=Ani	32,65	25,94	41,41
A=Sce B=Spa C=Smi	84,01	7,36	8,63
A=Sce-Spa B=Smi C=Sku	72,83	11,88	11,88
A=Sce-Spa-Smi B=Sku C=Sba	52,93	15,68	31,39
A=Sce-Spa-Smi-Sku B=Sba C=Sca	99	0,09	0,91
A=Sce-Spa-Smi-Sku-Sba B=Sca C=Cgl	36,35	35,73	27,92
A=Sce-Spa-Smi-Sku-Sba-Sca B=Cgl C=Kpo	46,08	24,09	29,84
A=Sce-Spa-Smi-Sku-Sba-Sca-Cgl-Kpo-Ago-Kla-Kwa-Skl B=Cal-Cdu-Ctr-Lel-Pst-Dha-Cgu-Clu C=Yli	90,08	3,59	6,33

Supplementary table 2 (2 of 6)

Topologies 4	(((A,B),C),D)	(((A,B),D),C)	(((A,C),B),D)	(((A,C),D),B)	(((A,D),B),C)	(((A,D),C),B)	(((B,C),A),D)	(((B,C),D),A)	(((B,D),A),C)	(((B,D),C),A)	(((C,D),A),B)	(((C,D),B),A)	((A,B),(C,D))	((A,C),(B,D))	((A,D),(B,C))
A=Fox-Fve-Gze-Nha<br>B=Tre<br>C=Mgr<br>D=Ncr-Cgo-Pan	18,29	18,03	0,14	0,35	0,31	0,07	0,7	0,47	0,54	0,31	1,18	0,83	58,28	0,23	0,29
A=Fox-Fve-Gze-Nha-Tre<br>B=Mgr-Ncr-Cgo-Pan<br>C=Bci<br>D=Ssc	0	0	0	0	0	0	0	0	0	0	4,72	5,8	89,48	0	0
A=Lbi<br>B=Cci<br>C=Pch<br>D=Ppl	7,08	14,32	0,63	0,75	1,03	0,72	0,1	0,38	0,94	0,17	9,73	2,65	60,98	0,22	0,3
A=Lbi-Cci-Pch-Ppl-Cne<br>B=Uma<br>C=Sro<br>D=Pgr	2,97	1,91	2,55	2,05	1,02	1,26	3,47	4,13	1,46	3,71	20,19	22,27	28,16	2,33	2,52
A=Ago<br>B=Kla<br>C=Kwa<br>D=Skl	7,52	10,64	1,72	4,56	1,77	5,03	1,31	3,04	2,04	2,85	10,61	7,55	36,33	2,4	2,63
A=Aor-Afl-Ate-Ang<br>B=Ani<br>C=Acl<br>D=Afu-Nfi	1,82	1,75	0,35	1,54	0,14	1,01	0,27	1	0,53	0,76	48,3	15,05	26,86	0,56	0,05
A=Aor-Afl-Ate-Ang-Ani<br>B=Acl-Afu-Nfi<br>C=Hca<br>D=Cim-Ure	9,36	8,22	0	0	0	0,29	0,15	0	0	0,15	1,46	1,1	79,28	0	0
A=Aor-Afl-Ate-Ang-Ani-Acl-Afu-Nfi<br>B=Hca-Cim-Ure<br>C=Sno<br>D=Mfi	10,07	8,98	0,13	0,03	0,39	0	0,46	0	0,07	0,13	0,85	0,72	78,04	0,13	0
A=Aor-Afl-Ate-Ang-Ani-Acl-Afu-Nfi-Hca-Cim-Ure<br>B=Sno-Mfi<br>C=Bci-Ssc<br>D=Mgr-Ncr-Cgo-Pan-Tre-Gze-Fox-Fve-Nha	6,74	1,09	2,26	1,72	0,47	1	2,78	2,88	0,49	1,46	21,84	23,97	31,17	1,32	0,81
A=Sce-Spa-Smi-Sku-Sba-Sca-Cgl<br>B=Kpo<br>C=Ago-Kla<br>D=Kwa-Skl	8,96	18,14	0,2	0,36	0,85	0,76	1,16	0,78	1,14	0,83	5,27	4,77	55,78	0,48	0,53
A=Sce-Spa-Smi-Sku-Sba-Sca-Cgl-Kpo<br>B=Ago-Kla-Kwa-Skl<br>C=Cal-Cdu-Ctr-Lel-Pst-Dha-Cgu<br>D=Clu	0,21	0,58	0	0	0	0	0	0,1	0	0	0	0,14	98,97	0	0,07
A=Sce-Spa-Smi-Sku-Sba-Sca-Cgl-Kpo-Ago-Kla-Kwa-Skl-Cal-Cdu-Ctr-Lel-Pst-Dha-Cgu-Clu<br>B=Yli<br>C=Aor-Afl-Ate-Ang-Ani-Acl-Afu-Nfi-Hca-Cim-Ure-Sno-Mfi<br>D=Bci-Ssc-Mgr-Ncr-Cgo-Pan-Tre-Gze-Fox-Fve-Nha	0,17	0,34	0	0	0	0	0,04	0,22	0,07	0	1,69	3,67	93,71	0	0,07
A=Sce-Spa-Smi-Sku-Sba-Sca-Cgl-Kpo-Ago-Kla-Kwa-Skl-Cal-Cdu-Ctr-Lel-Pst-Dha-Cgu-Clu-Yli<br>B=Aor-Afl-Ate-Ang-Ani-Acl-Afu-Nfi-Hca-Cim-Ure-Sno-Mfi-Bci-Ssc-Mgr-Ncr-Cgo-Pan-Tre-Gze-Fox-Fve-Nha<br>C=Sja-Spb<br>D=Pca	8,27	9,6	6,06	2,91	4,11	1,29	2,39	0,86	5,01	3,63	7,21	8,55	34	4,68	1,43
A=Sce-Spa-Smi-Sku-Sba-Sca-Cgl-Kpo-Ago-Kla-Kwa-Skl-Cal-Cdu-Ctr-Lel-Pst-Dha-Cgu-Clu-Yli-Aor-Afl-Ate-Ang-Ani-Acl-Afu-Nfi-Hca-Cim-Ure-Sno-Mfi-Bci-Ssc-Mgr-Ncr-Cgo-Pan-Tre-Gze-Fox-Fve-Nha<br>B=Sja-Spb-Pca<br>C=Cci-Lbi-Pch-Ppl-Cne-Sro-Pgr<br>D=Uma	1,08	1,15	0	0,29	0,59	0,44	0,07	0,59	0,49	0,29	3,46	2,8	88,73	0	0
A=Sce-Spa-Smi-Sku-Sba-Sca-Cgl-Kpo-Ago-Kla-Kwa-Skl-Cal-Cdu-Ctr-Lel-Pst-Dha-Cgu-Clu-Yli-Aor-Afl-Ate-Ang-Ani-Acl-Afu-Nfi-Hca-Cim-Ure-Sno-Mfi-Bci-Ssc-Mgr-Ncr-Cgo-Pan-Tre-Gze-Fox-Fve-Nha-Sja-Spb-Pca<br>B=Cci-Lbi-Pch-Ppl-Cne-Sro-Pgr-Uma<br>C=Bde<br>D=Pbl-Ror	5,01	28,37	1,23	1,24	3,04	0,99	1,44	1,89	6,51	4,47	5,35	16,23	22,16	0,86	1,2

Supplementary table 2 (3 of 6)

T21 phylome

Topologies 3	((A,B),C)	((A,C),B)	((B,C),A)
A=Cal<br>B=Cdu<br>C=Ctr	97,69	0,89	1,43
A=Cal-Cdu<br>B=Ctr<br>C=Lel	89,91	5,12	4,98
A=Cal-Cdu-Ctr<br>B=Lel<br>C=Pst	90,02	5,88	4,11
A=Cal-Cdu-Ctr-Lel-Pst<br>B=Dha-Cgu<br>C=Clu	50,10	17,27	32,63
A=Sce<br>B=Spa<br>C=Smi	84,38	7,42	8,20
A=Sce-Spa<br>B=Smi<br>C=Sku	72,48	12,04	15,48
A=Sce-Spa-Smi<br>B=Sku<br>C=Sba	52,62	15,32	32,07
A=Sce-Spa-Smi-Sku<br>B=Sba<br>C=Sca	99,28	0,46	0,26
A=Sce-Spa-Smi-Sku-Sba<br>B=Sca<br>C=Cgl	34,90	36,24	28,87
A=Sce-Spa-Smi-Sku-Sba-Sca<br>B=Cgl<br>C=Kpo	52,66	24,67	22,66

Topologies 4	**(((A,B),C),D)**	**(((A,B),D),C)**	**(((A,C),B),D)**	**(((A,C),D),B)**	**(((A,D),B),C)**	**(((A,D),C),B)**	**(((B,C),A),D)**	**(((B,C),D),A)**	**(((B,D),A),C)**	**(((B,D),C),A)**	**(((C,D),A),B)**	**(((C,D),B),A)**	**((A,B),(C,D))**	**((A,C),(B,D))**	**((A,D),(B,C))**
A=Sce-Spa-Smi-Sku-Sba-Sca-Cgl<br>B=Kpo<br>C=Ago-Kla<br>D=Kwa-Skl	8,70	17,86	0,53	0,33	0,80	0,62	0,61	0,76	0,69	0,58	7,20	5,32	54,70	0,87	0,44
A=Sce-Spa-Smi-Sku-Sba-Sca-Cgl-Kpo<br>B=Ago-Kla-Kwa-Skl<br>C=Cal-Cdu-Ctr-Lel-Pst-Dha-Cgu<br>D=Clu	1,89	1,50	0,00	0,00	0,17	0,00	0,06	0,00	0,52	0,00	1,29	1,37	92,86	0,17	0,17
A=Ago<br>B=Kla<br>C=Kwa<br>D=Skl	9,29	12,46	2,74	4,88	1,99	4,64	2,05	3,61	2,26	2,06	5,13	6,85	36,05	2,71	3,28
A=Cal-Cdu-Ctr-Lel<br>B=Pst<br>C=Dha<br>D=Cgu	23,75	7,98	2,03	0,38	1,06	0,84	7,46	7,55	0,90	2,67	3,38	14,22	24,74	0,46	2,60

Supplementary table 2 (5 of 6)

T12a phylome

Topologies 3	((A,B),C)	((A,C),B)	((B,C),A)
A=Sce<br>B=Spa<br>C=Smi	84,58	7,21	8,21
A=Sce-Spa<br>B=Smi<br>C=Sku	72,89	12,13	14,98
A=Sce-Spa-Smi<br>B=Sku<br>C=Sba	52,86	14,98	32,15
A=Sce-Spa-Smi-Sku<br>B=Sba<br>C=Sca	98,96	0,65	0,66
A=Sce-Spa-Smi-Sku-Sba<br>B=Sca<br>C=Cgl	35,12	36,61	28,27
A=Sce-Spa-Smi-Sku-Sba-Sca<br>B=Cgl<br>C=Kpo	49,99	23,05	26,95

Topologies 4	(((A,B),C),D)	(((A,B),D),C)	(((A,C),B),D)	(((A,C),D),B)	(((A,D),B),C)	(((A,D),C),B)	(((B,C),A),D)	(((B,C),D),A)	(((B,D),A),C)	(((B,D),C),A)	(((C,D),A),B)	(((C,D),B),A)	((A,B),(C,D))	((A,C),(B,D))	((A,D),(B,C))
A=Ago<br>B=Kla<br>C=Kwa<br>D=Skl	9,15	10,86	2,28	5,73	1,91	5,19	0,87	3,62	2,08	2,28	9,91	5,06	35,85	2,53	2,69

T12b phylome

Topologies 3	**((A,B),C)**	**((A,C),B)**	**((B,C),A)**
A=Ago<br>B=Cal<br>C=Sce	1,58	96,52	1,9
A=Sce-Ago<br>B=Cal<br>C=Yli	87,14	4,5	8,36
A=Sce-Ago-Cal-Yli<br>B=Bci-Ncr-Sno-Ani<br>C=Spb	50,41	26,13	23,46
A=Sce-Ago-Cal-Yli-Bci-Ncr-Sno-Ani<br>B=Spb<br>C=Cne	60,02	21,29	48,69
A=Sce-Ago-Cal-Yli-Bci-Ncr-Sno-Ani-Spb<br>B=Cne<br>C=Ror	30,56	19,73	49,71

Topologies 4	**(((A,B),C),D)**	**(((A,B),D),C)**	**(((A,C),B),D)**	**(((A,C),D),B)**	**(((A,D),B),C)**	**(((A,D),C),B)**	**(((B,C),A),D)**	**(((B,C),D),A)**	**(((B,D),A),C)**	**(((B,D),C),A)**	**(((C,D),A),B)**	**(((C,D),B),A)**	**((A,B),(C,D))**	**((A,C),(B,D))**	**((A,D),(B,C))**
A=Bci<br>B=Ncr<br>C=Sno<br>D=Ani	23,8	17,21	5,26	4,5	2,8	3,07	2,44	1,33	2,32	0,75	7,95	2,38	22,14	1,98	2,07
A=Sce-Ago-Cal<br>B=Yli<br>C=Bci-Ncr<br>D=Sno-Ani	0,53	1,1	0	0	0,03	0	0,08	0	0	0,08	2,96	5,95	89,28	0	0
