# Supplementary material for: The Tree versus the Forest: The Fungal Tree of Life and the Topological Diversity within the Yeast Phylome
Source: PLoS One. 2009 Feb 3;4(2):e4357. doi: 10.1371/journal.pone.0004357 (PMC2629814; doi:10.1371/journal.pone.0004357)
Supplement: Table S3 — (0.19 MB PDF) [file pone.0004357.s009.pdf]

Table S3

Species included in each of the species trees based on randomly-sampled species.

Supplementary table 3

	Code	Organism name	T50a	T50b	T50c	T40a	T40b	T40c	T30a	T30b	T30c
Saccharomyces sensu stricto	Sce	Saccharomyces cerevisiae	■	■	■	■	■	■	■	■	■
	Spa	Saccharomyces paradoxus	■	■	■	■	■	■	■	■	■
	Smi	Saccharomyces mikatae	■	■	■	■	■	■	■	■	■
	Sku	Saccharomyces kudriavzevii	■	■	■	■	■				■
	Sba	Saccharomyces bayanus	■	■	■			■		■	
Saccharomyces complex	Sca	Saccharomyces castellii	■	■	■	■			■	■	
	Cgl	Candida glabrata	■		■	■	■	■		■	■
	Kpo	Kluyveromyces polysporus	■	■	■	■	■				■
	Ago	Ashbya gossypii	■	■	■	■	■			■	
	Kla	Kluyveromyces lactis	■	■	■			■			
	Kwa	Kluyveromyces waltii	■	■	■						
	Skl	Saccharomyces kluyveri			■	■	■	■	■		
Candida cluster	Cal	Candida albicans	■	■	■	■	■	■	■	■	
	Cdu	Candida dubliniensis	■	■	■	■		■	■		
	Ctr	Candida tropicalis	■	■	■		■	■	■	■	■
	Lel	Lodderomyces elongisporus	■	■	■	■	■		■		
	Pst	Pichia stipitis		■	■	■					
	Dha	Debaryomyces hansenii	■		■	■	■	■	■	■	■
	Cgu	Candida guilliermondii	■	■	■					■	
	Clu	Candida lusitaniae			■		■		■	■	■
Dipodascaceae	Yli	Yarrowia lipolytica	■	■	■		■	■		■	■
Leotiomycetes	Bci	Botrytis cinerea	■		■	■	■	■	■	■	
	Ssc	Sclerotinia sclerotiorum	■	■	■		■	■	■	■	
Sodariomycetes	Mgr	Magnaporthe grisea	■	■	■		■	■	■		■
	Ncr	Neurospora crassa	■	■	■	■	■	■	■		■
	Cgo	Chaetomium globosum	■	■		■		■			
	Pan	Podospora anserina	■	■	■		■	■			■
	Tre	Trichoderma reesei	■		■	■	■	■	■	■	
	Gze	Fusarium graminearum	■	■	■		■			■	■
	Fox	Fusarium oxysporum	■	■	■	■		■			■
	Fve	Fusarium verticillioides	■	■	■	■	■	■			■
	Nha	Nectria haematococca			■	■	■			■	
Dothideomycetes	Sno	Stagonospora nodorum		■		■	■	■	■		■
	Mfi	Mycosphaerella fijiensis	■	■	■	■					■
Eurotiomycetes	Hca	Histoplasma capsulatum	■	■	■		■	■	■		
	Cim	Coccidioides immitis	■	■	■	■	■	■	■	■	■
	Ure	Uncinocarpus reesii	■	■	■	■		■	■	■	
	Acl	Aspergillus clavatus	■	■		■	■		■	■	■
	Afu	Aspergillus fumigatus	■	■	■	■	■	■		■	■
	Nfi	Neosartorya fischeri	■	■	■	■	■		■		
	Ani	Aspergillus nidulans	■	■	■		■				
	Ang	Aspergillus niger	■	■	■	■		■		■	■
	Aor	Aspergillus oryzae	■	■	■		■		■	■	■
	Afl	Aspergillus flavus	■	■		■	■	■			
	Ate	Aspergillus terreus	■	■	■	■		■			■
Taphrinomycotina	Spb	Schizosaccharomyces pombe		■	■	■					
	Sja	Schizosaccharomyces japonicus	■	■		■	■	■			
	Pca	Pneumocystis carinii		■				■		■	
Basidiomycota	Cci	Coprinus cinereus	■	■	■		■		■		■
	Cne	Cryptococcus neoformans	■	■					■		
	Lbi	Laccaria bicolor	■	■	■	■		■	■	■	■
	Ppl	Postia placenta					■	■	■		
	Pch	Phanerochaete chrysosporium	■	■		■	■				
	Pgr	Puccinia graminis	■	■	■	■	■	■		■	
	Sro	Sporobolomyces roseus		■	■	■	■	■		■	■
	Uma	Ustilago maydis	■	■				■	■	■	■
Zygomycota	Ror	Rhizopus oryzae	■	■	■	■		■	■		
	Pbl	Phycomyces blakesleeanus	■		■		■	■			■
Chitridiomycota	Bde	Batrachochytrium dendrobatidis		■	■	■	■	■	■	■	
Microsporidia	Ecu	Encephalitozoon cuniculi	■		■	■	■		■		■
