# Supplementary material for: The Tree versus the Forest: The Fungal Tree of Life and the Topological Diversity within the Yeast Phylome
Source: PLoS One. 2009 Feb 3;4(2):e4357. doi: 10.1371/journal.pone.0004357 (PMC2629814; doi:10.1371/journal.pone.0004357)
Supplement: Table S4 — (0.14 MB PDF) [file pone.0004357.s010.pdf]

Table S4

List of yeast seed proteins included in each species tree, the specific alignments of these protein families can be accessed at phylomeDB (http://www.phylomedb.org).

Supplementary table 4 (1 of 11)

T60:

YGL095C, YDR460W, YLR005W, YDR449C, YNL061W, YOL021C, YGL233W, YCR042C, YDR083W, YAL036C, YOR330C, YPR088C, YNL072W, YLR277C, YFR009W, YMR236W, YLR129W, YBL023C, YGR200C, YDL164C, YER082C, YNL048W, YOR168W, YJL115W, YMR214W, YPL217C, YKR038C, YBR237W, YMR093W, YNR035C, YBR236C, YGL163C, YPR173C, YOR164C, YLR166C, YJR105W, YML096W, YHR200W, YAL039C, YBL035C, YOR119C, YIL022W, YGR006W, YJL074C, YGL087C, YDR196C, YJR005W, YGL078C, YDR211W, YMR301C, YMR078C, YPL001W, YBR234C, YDR087C, YKL139W, YLR195C, YMR288W, YBR166C, YLR409C, YHR188C, YCL030C, YGR261C, YHL004W, YFR010W, YOL062C, YDR331W, YFL046W, YKL021C, YDR080W

T21:

YPL157W, YJL104W, YJL106W, YJL130C, YJL091C, YDR361C, YFR009W, YJL111W, YML030W, YHR140W, YJL140W, YJL115W, YJL121C, YDR292C, YPL208W, YJL054W, YJL057C, YJL031C, YJL033W, YJL046W, YGR200C, YOL009C, YJL072C, YJL085W, YJL063C, YJL068C, YJL069C, YJR014W, YJR016C, YLR005W, YJR005W, YJR070C, YJR010W, YJL141C, YGR175C, YBR166C, YJR050W, YJR033C, YLR299W, YDR373W, YJL178C, YPL030W, YJL180C, YJR024C, YPR133C, YJL155C, YJL183W, YJR065C, YJL208C, YJR001W, YJL194W, YJL197W, YJL200C, YHR169W, YIL010W, YIL021W, YIL022W, YOR361C, YIL007C, YMR091C, YHR172W, YIL041W, YHL029C, YIL044C, YIL033C, YIL034C, YIL039W, YHR181W, YHR188C, YHR191C, YHR170W, YGR083C, YGR119C, YHR194W, YNL248C, YMR304W, YDR228C, YNR050C, YHR201C, YHR202W, YPL009C, YIL157C, YIR005W, YPL031C, YIL140W, YDR297W, YNL062C, YIR034C, YOR027W, YJL006C, YIR008C, YIR012W, YNL059C, YGL125W, YOL049W, YER162C, YIL062C, YIL063C, YIL064W, YIL093C, YJL081C, YIL125W, YIL128W, YIL096C, YPR066W, YIL106W, YKL078W, YKL188C, YKL190W, YKL194C, YJL012C, YKL182W, YKL184W, YKL197C, YBR101C, YGR076C, YKR017C, YPR055W, YKL212W, YNR035C, YPL217C, YKL139W, YKL143W, YOL018C, YPL132W, YKL135C, YPL093W, YGL130W, YKL170W, YKL179C, YKL149C, YKL155C, YCL054W, YGR163W, YLL018C, YNL313C, YER052C, YDL056W, YOR290C, YHR036W, YMR026C, YPR024W, YLR409C, YLL032C, YLL034C, YLR002C, YKR038C, YBR114W, YIL020C, YKR024C, YMR211W, YOR163W, YBR121C, YKL047W, YKR070W, YPR141C, YNL200C, YDL051W, YKR068C, YJR052W, YJR110W, YJR118C, YJR129C, YJR102C, YJR105W, YHR011W, YJR132W, YLR370C, YKL125W, YKL003C, YJR138W, YJR139C, YDR167W, YJR072C, YJR074W, YOR115C, YJR057W, YJR062C, YJR064W, YJR088C, YJR099W, YNL186W, YJR101W, YCR059C, YJR095W, YJR097W, YKL059C, YCL009C, YKL077W, YKL041W, YFL001W, YKL056C, YKL113C, YKL099C, YKL106W, YKL110C, YNL325C, YLR051C, YKL095W, YMR005W, YKL014C, YKL016C, YNL175C, YKL010C, YDR176W, YLR314C, YKL025C, YKL034W, YKL035W, YKL019W, YKL021C, YKL024C, YJL187C, YGL043W, YOR077W, YGL055W, YPR062W, YMR293C, YDL100C, YGL058W, YGL085W, YGL086W, YGL087C, YGL068W, YGL077C, YKR023W, YMR093W, YFR048W, YFR050C, YKR097W, YFR041C, YNL330C, YGL001C, YGL017W, YIR026C, YGL019W, YGL003C, YGL011C, YGL016W, YGL153W, YGL155W, YGL163C,

YGL137W, YGL142C, YAL039C, YML001W, YHR025W, YGL180W, YGL192W, YNL227C, YGL171W, YGL172W, YGL103W, YLR248W, YGL110C, YHR171W, YGL095C, YGL099W, YGL111W, YGR185C, YGL091C, YGL136C, YGL114W, YGL115W, YBR200W, YER012W, YFL028C, YER090W, YFL037W, YER080W, YER082C, YER087W, YER093C, YER133W, YER134C, YER140W, YER110C, YOR168W, YHR147C, YKL173W, YER023W, YNL022C, YER016W, YER020W, YPR187W, YLR253W, YER065C, YGR112W, YER072W, YLR143W, YER049W, YER055C, YFL027C, YFL029C, YGL129C, YFL008W, YFL016C, YDR214W, YHL015W, YGL119W, YFR021W, YFR028C, YER091C, YDR416W, YFL046W, YER164W, YER165W, YER173W, YER141W, YER152C, YER156C, YER174C, YMR276W, YFL002C, YKL114C, YDR531W, YER182W, YER183C, YGR229C, YNL219C, YFL034C-B, YHL019C, YGR276C, YGR285C, YHL004W, YHL030W, YHR007C, YHR024C, YHR028C, YHL031C, YHL032C, YHR004C, YGR252W, YGR253C, YGR255C, YGR231C, YGR240C, YGR244C, YGR258C, YGR270W, YJL074C, YGR274C, YGR261C, YGR264C, YNR015W, YDL167C, YHR132C, YHR134W, YPR026W, YHR114W, YHR120W, YCL059C, YGR061C, YGL233W, YKL206C, YHR144C, YHR150W, YHR151C, YGR110W, YNL099C, YHR059W, YBR187W, YHR038W, YPR168W, YDR041W, YHR076W, YKL017C, YHR088W, YHR069C, YHR072W, YHR075C, YGL194C, YEL026W, YGR028W, YGR031W, YGR005C, YGR006W, YDL112W, YJL084C, YGR080W, YGR090W, YGR103W, YGR046W, YMR150C, YGR078C, YGL201C, YCL034W, YOR124C, YBR082C, YHR166C, YGL200C, YGL221C, YGL244W, YOL124C, YGR001C, YGL234W, YGL238W, YGL241W, YGR187C, YGR194C, YGR195W, YGR174C, YGR178C, YGR186W, YJL059W, YGR215W, YDR235W, YKL028W, YGL141W, YGR208W, YGR210C, YLR312W-A, YKL193C, YGR144W, YHR045W, YBR234C, YML126C, YHL002W, YOR206W, YGR172C, YGR173W, YER086W, YGR155W, YHR205W, YGL207W, YOR125C, YOR128C, YLR362W, YER157W, YEL053C, YOR130C, YDL183C, YOR157C, YOR158W, YOR143C, YNL317W, YOR145C, YOR046C, YOR051C, YOR052C, YOR021C, YOR036W, YOR038C, YOR056C, YOR074C, YLR105C, YGL048C, YOR065W, YOR067C, YOR070C, YOR259C, YOR278W, YOR287C, YOR251C, YOR254C, YGL212W, YOR289W, YNL032W, YOR319W, YOR323C, YLL001W, YOR294W, YOR310C, YOR207C, YNL306W, YOR212W, YMR167W, YOR164C, YOR166C, YOR216C, YOR241W, YOR245C, YOR250C, YOR217W, YOR223W, YJL030W, YMR079W, YPR108W, YIL003W, YKR001C, YNR012W, YNR017W, YNL263C, YJR073C, YHR200W, YNR052C, YDR335W, YNR038W, YPR169W, YMR309C, YNL308C, YNL310C, YLL019C, YNL290W, YNL292W, YDR267C, YNL315C, YBL091C, YNR006W, YNR007C, YKL088W, YNL328C, YNL329C, YKL080W, YOL041C, YNL241C, YBR279W, YOL078W, YML110C, YOL098C, YOL140W, YOL142W, YNL223W, YGR233C, YMR201C, YOL135C, YGL231C, YAL009W, YIL083C, YOL008W, YOL012C, YOL022C, YER125W, YOL067C, YHR193C, YOL076W, YGL047W, YOL061W, YOL062C, YNL291C, YGL236C, YPR048W, YPR049C, YPR029C, YPR031W, YPR041W, YMR015C, YIL097W, YLR085C, YPR069C, YKL205W, YPR060C, YPR061C, YBR073W, YOR261C, YPL266W, YNL128W, YPL237W, YPL239W, YPL268W, YMR218C, YGL213C, YLR075W, YPL270W, YPR010C, YHL013C, YPR143W, YML121W, YPR162C, YPR135W, YPR140W, YKR079C, YJR122W, YPR179C, YPR180W, YDL031W, YOR095C, YNR039C, YPR175W, YPR082C, YPL147W, YPR088C, YGL040C, YPR073C, YPL023C, YOR017W, YPR110C, YPR113W,

YLR396C, YPR097W, YJL101C, YJL011C, YOR326W, YPL029W, YPL115C, YOR288C, YIL153W, YPL012W, YJR068W, YPL046C, YPL083C, YPL084W, YPL086C, YPL051W, YOR119C, YNL006W, YOR336W, YOR358W, YNR032W, YOR330C, YOR332W, YMR127C, YIL090W, YPL002C, YPR167C, YIL142W, YOR370C, YGL245W, YPL001W, YPL172C, YPL228W, YMR288W, YHR042W, YPL169C, YPL170W, YOR252W, YPL211W, YPL214C, YPL215W, YDL006W, YPL195W, YPL209C, YOR211C, YPL107W, YOL065C, YPL101W, YPL103C, YMR239C, YPL116W, YPL133C, YPL151C, YPL160W, YPL117C, YPL128C, YPL131W, YLR397C, YJL124C, YML036W, YML038C, YML020W, YDR359C, YOR004W, YML042W, YML060W, YMR266W, YML069W, YML046W, YML049C, YLR378C, YLR435W, YLR439W, YLR440C, YOR001W, YLR419W, YLR427W, YLR452C, YML004C, YML008C, YKL013C, YAR003W, YGL167C, YML002W, YMR002W, YMR009W, YGL243W, YML127W, YML130C, YMR001C, YMR012W, YBR282W, YPR131C, YMR060C, YMR013C, YMR027W, YJR090C, YML092C, YML093W, YCR032W, YAL044C, YML080W, YBL104C, YML096W, YML102W, YML105C, YML115C, YML097C, YDR175C, YML099C, YLR008C, YLR129W, YBR103W, YKR016W, YLR084C, YLR103C, YPR160W, YER007W, YDR306C, YOL023W, YLR197W, YMR277W, YDR470C, YHR018C, YLR022C, YCR092C, YDR159W, YIL145C, YDL108W, YLR017W, YLR208W, YLR059C, YPR021C, YLR078C, YKR065C, YKL092C, YJR063W, YDR390C, YLR290C, YJR042W, YOL125W, YLR272C, YNL113W, YMR212C, YLR345W, YOR275C, YGR123C, YNL279W, YKR083C, YLR330W, YGL097W, YKL085W, YBR260C, YLR200W, YLR201C, YLR205C, YLR229C, YLR240W, YMR260C, YDL063C, YNL061W, YNL218W, YLR239C, YNL048W, YER078C, YKL189W, YNL119W, YBR159W, YNL110C, YNL111C, YGR095C, YER155C, YNL139C, YJR113C, YIL065C, YPL045W, YDR489W, YNL063W, YNR029C, YNL071W, YBR243C, YNL201C, YIR006C, YNL072W, YNL097C, YNL168C, YNL102W, YNL073W, YNL082W, YNL084C, YNL250W, YNL252C, YPL097W, YNL229C, YNL036W, YKL002W, YNL256W, YNL272C, YDR430C, YNL277W, YNL132W, YNL260C, YNL261W, YGR205W, YNL181W, YBR053C, YNL151C, YNL153C, YDR117C, YLR361C, YNL221C, YNL222W, YFL038C, YGR104C, YNL088W, YOR262W, YMR061W, YMR197C, YGL078C, YMR213W, YMR154C, YKR035W-A, YER068W, YMR214W, YMR229C, YMR231W, YMR236W, YPR019W, YBR162C, YMR224C, YLR418C, YMR092C, YFR045W, YMR062C, YMR077C, YNL287W, YIL134W, YNL075W, YMR146C, YDL164C, YMR193W, YOR334W, YMR129W, YNL005C, YNL010W, YGR152C, YMR297W, YMR308C, YMR315W, YNL016W, YPR016C, YML035C, YDL153C, YER041W, YNL023C, YNL025C, YNL312W, YMR263W, YNL137C, YMR240C, YMR241W, YEL058W, YMR278W, YMR290C, YJL062W, YMR296C, YKL058W, YMR287C, YOR117W, YHR073W, YCL039W, YDR158W, YBR039W, YBR135W, YDL116W, YBR154C, YLR015W, YDR484W, YDR257C, YBR151W, YGR007W, YML094W, YDR244W, YDL156W, YDR449C, YDR238C, YDL119C, YPR047W, YMR216C, YDR245W, YBR123C, YDL201W, YDR405W, YBR185C, YDR414C, YDL090C, YDL087C, YDR296W, YDL014W, YBR196C, YOR196C, YDR266C, YBR192W, YMR223W, YOL145C, YDL107W, YGL169W, YBR160W, YDL097C, YLR262C, YDR265W, YBR276C, YBR170C, YGL037C, YBR061C, YBR246W, YBR041W, YBR060C, YDL160C, YLR026C, YPR103W, YDR232W, YHR148W, YDR211W, YDR212W, YDR496C, YDR493W, YOR346W, YBR038W, YGR057C, YCL052C, YBR058C, YDR204W, YGL012W, YOL054W, YDR487C,

YBR095C, YDR454C, YAL034W-A, YDR459C, YDR231C, YLR133W, YDL132W, YPR183W, YDR234W, YPL126W, YDR236C, YBR087W, YDR468C, YJL005W, YDR473C, YDR472W, YHR111W, YBR094W, YDR460W, YBR088C, YDR465C, YIL002C, YOR232W, YDR339C, YDR301W, YLR285W, YGR145W, YCL031C, YHR142W, YCR003W, YMR247C, YBR056W, YDR337W, YCL055W, YOR271C, YDR101C, YDL064W, YPL243W, YDR365C, YDL004W, YML021C, YDL001W, YCL017C, YHR013C, YDR298C, YDR322W, YCR042C, YCR087C-A, YDR328C, YCR036W, YCR047C, YBR261C, YCR051W, YPL104W, YOR360C, YHR204W, YCR008W, YKL033W, YNR054C, YCR088W, YDR448W, YKR080W, YOR175C, YDR190C, YOR165W, YDR331W, YCR079W, YMR301C, YBR229C, YML059C, YLR289W, YDL065C, YBR237W, YBR249C, YDR311W, YDR270W, YGR193C, YBR236C, YDR308C, YAR015W, YBR198C, YDR397C, YPR105C, YDL072C, YBR217W, YBR220C, YBL037W, YPR067W, YMR125W, YOL077C, YBR281C, YBR251W, YBR271W, YDR376W, YER043C, YBR193C, YDR371W, YDL040C, YBR286W, YBR290W, YBR252W, YGL154C, YDR208W, YBR248C, YDR280W, YBR265W, YBR267W, YBR269C, YMR314W, YBR254C, YLR222C, YDR196C, YDR092W, YOL032W, YDL122W, YEL056W, YBL076C, YDL212W, YDR097C, YDR036C, YPR137W, YDL234C, YOR340C, YAL022C, YIL043C, YDR165W, YBL057C, YCL016C, YPR139C, YPL120W, YOR014W, YJR006W, YKL211C, YDR177W, YKR014C, YOL040C, YML098W, YOR198C, YBL011W, YER003C, YOL010W, YGL026C, YDR170C, YAL041W, YEL032W, YEL037C, YFR052W, YEL062W, YJR140C, YDR083W, YNL161W, YDR006C, YBL033C, YDR002W, YEL038W, YBL038W, YNR003C, YDR087C, YEL024W, YDR023W, YDR080W, YBL055C, YBL052C, YCR053W, YGR272C, YEL036C, YGR171C, YAL036C, YDL111C, YDL143W, YBL023C, YDR529C, YBR011C, YER006W, YDR049W, YDR120C, YDR189W, YMR208W, YGR149W, YER005W, YAL001C, YIL035C, YDR188W, YDL185W, YMR010W,YEL050C, YBR029C, YOR111W, YDR523C, YGR262C, YDR121W, YMR078C, YCR072C, YDR527W, YAR018C, YAR007C, YCL030C, YOL093W, YDR152W, YDL198C, YDR142C, YGL065C, YEL051W, YBL105C, YBR002C, YBR003W, YAL010C, YMR171C, YEL029C, YJL087C, YDL205C, YDR108W, YDL207W, YBL095W, YDL215C, YBL007C, YER009W, YDR140W, YOR035C, YDR047W, YAL002W, YDL190C, YDR067C

T12a:

YLR079W, YDL142C, YLR074C, YGL099W, YLR299W, YGR212W, YLR022C, YJL057C, YLR059C, YLR283W, YDR067C, YLR105C, YOR249C, YLR129W, YLR085C, YLR084C, YLR103C, YPL004C, YLR015W, YLR008C, YLR021W, YCL039W, YLR005W, YKR002W, YLR007W, YLR060W, YLR057W, YLR052W, YLR051C, YOL019W, YGL213C, YDR339C, YIL002C, YLR033W, YAL036C, YDR337W, YLR257W, YLR268W, YLR262C, YOR067C, YLR205C, YLR253W, YLR250W, YDL217C, YCR072C, YLR290C, YLR305C, YLR077W, YIL161W, YLR285W, YMR203W, YLR288C, YLR199C, YKR080W, YBR131W, YLR023C, YLR143W, YKL017C, YLR172C, YBL052C, YDL006W, YLR229C, YDR253C, YLR239C, YML052W, YBR286W, YOR288C, YLR213C, YLR211C, YKL211C, YKL209C, YKR001C, YLR034C, YGL037C, YKL179C, YHR069C, YOR360C, YDR080W, YDR484W, YDR158W, YMR193W, YIL097W, YER006W, YKR006C, YKR011C, YKR008W, YMR229C, YKL171W, YKL176C, YKL175W, YKL159C, YJR005W, YKL170W, YDR262W, YKL197C, YKL191W,

YKL190W, YKL194C, YLR272C, YJR080C, YML110C, YOR286W, YKL186C, YKR099W, YDR466W, YLL011W, YLL001W, YDR235W, YDL063C, YGL058W, YKR090W, YLR002C, YLL032C, YLL029W, YLL038C, YPR110C, YLL019C, YLL018C, YLL027W, YNR006W, YKL087C, YOR184W, YKR065C, YNL107W, YOR257W, YKR024C, YKR037C, YNL108C, YKR086W, YLR197W, YKR079C, YKR084C, YKR083C, YKL122C, YKR068C, YKR071C, YKR070W, YMR002W, YJR074W, YDR335W, YLR017W, YDL178W, YJL016W, YML097C, YOR353C, YML121W, YBR084W, YMR014W, YMR012W, YMR021C, YMR015C, YDR183W, YDL198C, YGL087C, YMR009W, YML092C, YER075C, YML096W, YOR104W, YDR256C, YLR412W, YDR201W, YML080W, YIR034C, YHR045W, YML105C, YBR228W, YML108W, YOR219C, YML098W, YML104C, YML102W, YDR523C, YAL001C, YPR183W, YMR098C, YMR087W, YMR061W, YGL019W, YMR091C, YOR335C, YGL086W, YMR126C, YMR135C, YMR128W, YMR107W, YOR157C, YGL228W, YPL151C, YDR440W, YMR044W, YMR060C, YMR054W, YMR027W, YPL084W, YMR041C, YMR029C, YMR083W, YMR077C, YJR017C, YGR251W, YMR079W, YBR133C, YMR062C, YMR067C, YMR065W, YLR398C, YLR397C, YLR409C, YIL005W, YLR385C, YOR141C, YLR396C, YDL212W, YML059C, YCL017C, YLR423C, YLR435W, YLR429W, YGR004W, YLR418C, YLR422W, YLR421C, YPL210C, YLR330W, YLR345W, YLR335W, YER069W, YLR309C, YLR320W, YLR314C, YLR378C, YLR368W, YJR068W, YGL231C, YLR369W, YKR097W, YLR352W, YNL230C, YLR359W, YER007C-A, YML046W, YML055W, YML049C, YBR039W, YML004C, YML042W, YML041C, YML076C, YPL011C, YML069W, YML072C, YML071C, YDR279W, YML060W, YML066C, YML064C, YML001W, YDL080C, YJR084W, YML002W, YGL092W, YPR004C, YLR447C, YLR443W, YML036W, YML029W, YDR175C, YML032C, YNL026W, YML014W, YML008C, YML021C, YML020W, YJL030W, YOR361C, YER012W, YJL031C, YJL019W, YIR017C, YFR048W, YJL023C, YGL205W, YJL053W, YJL049W, YGR078C, YJL054W, YML048W, YJL042W, YPL115C, YJL045W, YIR008C, YIR007W, YCR079W, YIR010W, YPL118W, YPR066W, YKL182W, YIR005W, YML120C, YJL011C, YER086W, YJL013C, YJL012C, YML114C, YIR026C, YJL006C, YIL038C, YJL115W, YDL078C, YDR165W, YJL117W, YJL104W, YJL074C, YJL111W, YJL106W, YJL147C, YDL176W, YJL141C, YJL146W, YJL145W, YDR403W, YJL124C, YJL140W, YGR030C, YJL068C, YJL066C, YNR035C, YER161C, YJL060W, YHL029C, YJL063C, YJL061W, YEL019C, YJL091C, YEL038W, YJL101C, YJL095W, YJL085W, YJL081C, YJL089W, YJL087C, YIL035C, YNL260C, YIL041W, YIL039W, YIL023C, YIL003W, YDL103C, YBR185C, YIL139C, YMR150C, YOL023W, YPR057W, YIL063C, YHR200W, YML130C, YJL157C, YIL046W, YMR114C, YHR205W, YCR087C-A, YHR207C, YAR014C, YIL044C, YMR232W, YHR202W, YIL022W, YIL017C, YDR318W, YOL104C, YNL099C, YIL007C, YLR399C, YHR017W, YOR368W, YPR137W, YIL126W, YDR170C, YIL129C, YIL116W, YHR028C, YIL125W, YPL072W, YLR287C, YIL155C, YIL153W, YHR201C, YIL157C, YIL144W, YIL140W, YIL084C, YIL147C, YIL076W, YCR003W, YIL083C, YPL243W, YIL071C, YOR198C, YGL145W, YIL072W, YIL115C, YIL098C, YKL173W, YIL112W, YNL290W, YJL183W, YNL312W, YIL096C, YIL093C, YKL028W, YKL024C, YKL021C, YKL027W, YMR263W, YKL016C, YJR136C, YKL019W, YLR137W, YKL125W, YKL047W, YKL045W, YKL049C, YKL048C, YBR103W, YKL033W, YKL041W, YKL035W, YHL002W, YJR129C, YOR206W, YJR134C, YJR125C, YJR024C,

YGR244C, YJR126C, YMR080C, YMR265C, YKL003C, YKL013C, YKL011C, YJR139C, YGL047W, YKL002W, YKL135C, YKL120W, YKL113C, YKL124W, YLR289W, YKL106W, YKL085W, YKL110C, YKL108W, YKL155C, YNL252C, YDR328C, YPL180W, YKL149C, YKL137W, YKL134C, YKL142W, YKL139W, YKL077W, YKL069W, YKL082C, YKL080W, YKL058W, YML099C, YGL167C, YKL059C, YPL208W, YPL024W, YHL015W, YKL103C, YKL101W, YKL088W, YEL029C, YKL092C, YKL090W, YJR010W, YML015C, YMR075W, YJR014W, YJR001W, YJL184W, YMR097C, YJL039C, YDR270W, YJR050W, YGR285C, YMR284W, YJR052W, YPL112C, YJR033C, YJR046W, YJR042W, YJL178C, YJL173C, YBL093C, YJL180C, YJL149W, YIL142W, YMR140W, YJL155C, YPL268W, YPR084W, YJL197W, YJL204C, YJL203W, YOR026W, YJL187C, YJL194W, YJL192C, YER172C, YJR099W, YGL004C, YJR102C, YJR095W, YDR375C, YOR001W, YJR096W, YJR124C, YER068W, YJR117W, YLR240W, YBR245C, YJR109C, YJR107W, YJR113C, YJR111C, YJR065C, YPL255W, YMR278W, YJR067C, YGR255C, YJR057W, YGR084C, YJR060W, YJR092W, YJR088C, YML003W, YHR166C, YPR018W, YML127W, YJR073C, YKL184W, YJR077C, YOR329C, YOR326W, YGL014W, YOR332W, YOR310C, YOR278W, YML031W, YOR311C, YLR200W, YOR349W, YOR346W, YOR354C, YNL309W, YOR340C, YDR244W, YDL207W, YOR341W, YDL122W, YOR271C, YOR276W, YOR275C, YPL263C, YOR128C, YGL195W, YOR265W, YOR298W, YOR290C, YOR289W, YPL075W, YOR294W, YNL151C, YGR083C, YOR287C, YKL188C, YCR068W, YNL075W, YNL054W, YPL031C, YGL095C, YMR247C, YPL028W, YKL099C, YOR298C-A, YPL069C, YPL066W, YIL124W, YPL070W, YPL051W, YLR361C, YHR206W, YPL059W, YBR255W, YHR140W, YPR088C, YOR370C, YLR133W, YBR235W, YJL029C, YKL206C, YPL016W, YPL009C, YPL007C, YPL015C, YGR184C, YPL002C, YPL001W, YPL005W, YJR110W, YOR124C, YOR122C, YAL002W, YOR125C, YOR116C, YHR124W, YDL051W, YOR117W, YOR241W, YOR145C, YOR144C, YOR158W, YPL253C, YLR427W, YOR130C, YOR143C, YDL111C, YOR069W, YMR001C, YCR061W, YOR070C, YOR051C, YNL077W, YDR459C, YOR056C, YPL147W, YML093W, YOR095C, YOR111W, YOR106W, YOR077W, YOR075W, YOR091W, YEL037C, YPL157W, YOR220W, YGL175C, YOR052C, YNL139C, YOR195W, YKL056C, YOR217W, YOR261C, YBR229C, YOR252W, YGR123C, YBR166C, YGL130W, YOR243C, YOR250C, YMR218C, YLR370C, YOR168W, YOR194C, YGR263C, YPR105C, YOR163W, YOR166C, YOR165W, YOR215C, YNL081C, YOR208W, YGL115W, YOR211C, YKL078W, YOR196C, YOR207C, YJR135C, YIL042C, YPR060C, YIL064W, YPR062W, YPR061C, YNR003C, YGR194C, YGL111W, YPR049C, YPR162C, YPR079W, YPR075C, YPR086W, YDR063W, YPR069C, YPR067W, YFR009W, YPR072W, YPR021C, YPR019W, YNL006W, YPR023C, YLR439W, YKR088C, YEL062W, YPR016C, YPR047W, YPR034W, YPR033C, YPR041W, YDL117W, YPR029C, YPR026W, YPR031W, YGR028W, YPR148C, YBL038W, YPR160W, YDL126C, YPR139C, YGR110W, YOL010W, YPR140W, YPR188C, YPR180W, YPR179C, YPR186C, YPR185W, YPR168W, YNL294C, YOR079C, YPR169W, YPR103W, YBL034C, YPR108W, YOR164C, YPR089W, YAL039C, YMR026C, YPR091C, YIL128W, YPR131C, YKR095W, YPR135W, YPR133C, YPR113W, YPR112C, YPR122W, YPR114W, YPL131W, YCR071C, YPL133C, YPL132W, YPL120W, YPL101W, YPL126W, YPL125W, YPL239W, YPL152W, YPL060W, YHR072W, YAL010C, YPL030W, YPL138C, YPL150W, YPL149W, YPL096W,

YDR496C, YPL098C, YPL097W, YPL083C, YPL078C, YPL085W, YPR097W, YHR188C, YJL046W, YBR281C, YPL117C, YPL116W, YPL104W, YPL103C, YNL039W, YJR138W, YPL217C, YPL215W, YPL228W, YPL222W, YKL025C, YPL183C, YBR217W, YPL213W, YPL270W, YOR262W, YPL260W, YDR297W, YJL208C, YDR460W, YPL241C, YMR106C, YPL252C, YPL177C, YPL173W, YPL181W, YOR025W, YPL164C, YPL161C, YPL172C, YPL169C, YPL211W, YKL104C, YGL233W, YOL057W, YPL209C, YPL193W, YLR201C, YPL195W, YPL194W, YNL073W, YNL072W, YNL076W, YPL029W, YDL112W, YNL032W, YNL071W, YBL023C, YNL123W, YNR015W, YNL085W, YMR216C, YNL097C, YPR024W, YOR209C, YNL084C, YNL083W, YNL023C, YNL022C, YML030W, YNL025C, YNL012W, YDL185W, YJL059W, YNL016W, YNL062C, YPL045W, YNL048W, YNL061W, YNL059C, YPL107W, YNL036W, YNL045W, YNL042W, YNL164C, YGR195W, YNL168C, YNL165W, YNL152W, YNL119W, YNL161W, YNL153C, YGL137W, YNL202W, YNL200C, YNL213C, YNL207W, YNL180C, YNL175C, YJR119C, YNL181W, YNL111C, YNL110C, YFL008W, YNL112W, YNL102W, YDL101C, YKR035W-A, YGR155W, YOR283W, YGL065C, YNL132W, YNL147W, YNL138W, YMR223W, YNL121C, YNL129W, YNL128W, YMR214W, YMR213W, YMR217W, YIL020C, YMR208W, YMR167W, YMR212C, YMR209C, YMR302C, YMR231W, YKR017C, YMR235C, YDR097C, YMR157C, YMR220W, YMR224C, YNL169C, YMR221C, YJR016C, YMR163C, YMR211W, YMR145C, YMR144W, YMR154C, YMR146C, YKR069W, YMR197C, YKR023W, YMR202W, YMR201C, YMR179W, YNL317W, YMR188C, YMR185W, YMR297W, YMR296C, YBL015W, YMR298W, YOR008C, YPL212C, YMR293C, YMR291W, YNL010W, YNL002C, YMR315W, YPR175W, YDR532C, YDR473C, YMR304W, YMR314W, YMR313C, YMR257C, YOR374W, YMR260C, YMR259C, YMR239C, YOL146W, YDR023W, YMR240C, YMR285C, YLR362W, YMR277W, YJR053W, YMR282C, YMR267W, YMR266W, YMR276W, YBR028C, YOL094C, YPL099C, YNR001C, YOL093W, YPL214C, YOL065C, YDR465C, YOL076W, YOL147C, YPL179W, YIL021W, YBR122C, YOL078W, YOL117W, YDL113C, YGR274C, YOL100W, YGL243W, YBL033C, YAL041W, YOL027C, YIL061C, YNL308C, YNL279W, YOL018C, YOL012C, YOL064C, YOL054W, YOL049W, YOL062C, YBR058C, YOL032W, YOL031C, YHL031C, YPL170W, YPL237W, YOR014W, YOR023C, YOR021C, YOR004W, YKL146W, YDR176W, YFR031C, YOR048C, YOR039W, YOR038C, YCL030C, YKL154W, YOR035C, YOR027W, YOR037W, YGL043W, YOL132W, YDL165W, YOL135C, YDL166C, YJL072C, YBL074C, YOL126C, YOL125W, YJR097W, YOL145C, YOL142W, YOL067C, YDL230W, YOL138C, YOL137W, YNL219C, YOL139C, YDR212W, YNL272C, YER015W, YCR047C, YNL261W, YDR362C, YGL178W, YNL263C, YNR039C, YPR167C, YNL292W, YNL306W, YNL304W, YNL287W, YNL281W, YNL291C, YIL106W, YNL229C, YNL224C, YNL233W, YCL008C, YOL140W, YNL218W, YER148W, YDL156W, YIL034C, YNL254C, YBR287W, YNL256W, YBR017C, YNL248C, YFL005W, YNL251C, YNL250W, YNR032W, YNR029C, YNR038W, YOL124C, YNL088W, YJL033W, YGR235C, YNR017W, YOL009C, YBL055C, YNR054C, YOL008W, YOL005C, YBR198C, YNR043W, YNR052C, YNR051C, YNL313C, YIL088C, YMR171C, YNL315C, YPR141C, YDR034C, YNL310C, YOR351C, YNR012W, YLL026W, YPR048W, YOR115C, YNR007C, YNL329C, YNL328C, YOL077C, YDR309C, YDR186C, YKL172W, YKR014C, YDR188W, YDR179C, YOL061W, YDR087C,YDR181C, YDR202C, YGL068W, YDR288W, YDR204W, YDR194C,

YGR111W, YDR359C, YPR082C, YDR177W, YDR152W, YMR155W, YDR189W, YDR155C, YDR137W, YDR489W, YDR142C, YDR140W, YIL134W, YDR167W, YMR287C, YML023C, YDR163W, YDR159W, YJL121C, YDR164C, YJR106W, YDR267C, YDR280W, YML061C, YDR205W, YDR257C, YHR144C, YDR265W, YGR173W, YDR301W, YDR493W, YDR307W, YDR292C, YKL167C, YPL269W, YDR296W, YML078W, YDR216W, YDR214W, YDR229W, YDR228C, YDR208W, YDR207C, YNR050C, YDR211W, YCR059C, YDL055C, YDL074C, YDR245W, YGR200C, YDR231C, YDR236C, YBR062C, YDR130C, YDL234C, YDL231C, YOR319W, YDL235C, YDL225W, YDL216C, YBL045C, YDL226C, YMR241W, YDR021W, YNL307C, YDR028C, YDR004W, YDR002W, YGR145W, YER140W, YDL215C, YGL003C, YIL043C, YDL183C, YDL180W, YER020W, YBR223C, YJL143W, YDL167C, YDL205C, YDL203C, YLR387C, YJR122W, YDL190C, YOR358W, YDL201W, YMR004W, YDR092W, YDR089W, YDR101C, YBR138C, YDR084C, YDR083W, YDR088C, YDR182W, YDR118W, YDR117C, YDR121W, YBR094W, YDR108W, YDR103W, YDR116C, YJR041C, YDR082W, YDR054C, YDR052C, YJL200C, YDR057W, YDR041W, YKL144C, YGR020C, YDR047W, YDR079W, YDR076W, YPR143W, YJL062W, YDR065W, YDR064W, YIL075C, YLR107W, YER077C, YER173W, YKR043C, YNL082W, YEL036C, YMR068W, YJL102W, YEL026W, YEL024W, YEL050C, YER087W, YEL053C, YEL051W, YBR271W, YJL090C, YEL046C, YEL043W, YEL015W, YDR499W, YDR495C, YDR520C, YGL101W, YMR290C, YDR487C, YDR494W, YDR308C, YEL001C, YNL005C, YEL009C, YEL005C, YDR527W, YJR006W, YDR531W, YDR529C, YER042W, YER040W, YER048C, YER043C, YER023W, YER049W, YER038C, YER027C, YLR312W-A, YJR118C, YML091C, YER072W, YER052C, YER022W, YAL055W, YER055C, YER021W, YER003C, YJR090C, YKR007W, YER005W, YEL056W, YIL062C, YEL061C, YGL250W, YER016W, YNL323W, YOL133W, YER017C, YER007W, YJL044C, YER128W, YER008C, YDR486C, YDR371W, YHR082C, YDR374C, YDR373W, YDR363W, YMR058W, YDR365C, YDL040C, YDR397C, YDR390C, YBR114W, YDR400W, YER093C, YFR001W, YJR070C, YDR386W, YDR361C, YDR331W, YDR036C, YDR334W, YDR332W, YDR315C, YDR311W, YDR322W, YIL016W, YOR279C, YDR354W, YFR040W, YDR357C, YHR194W, YML126C, YKL112W, YLR026C, YPL242C, YOR058C, YDR468C, YDR464W, YDR449C, YFL040W, YDR456W, YDR414C, YDR481C, YDR477W, YDR485C, YHR176W, YDR472W, YDR470C, YDR476C, YNL063W, YDR446W, YDR049W, YDR416W, YDR425W, YIR012W, YDR411C, YDR407C, YDR415C, YDR454C, YMR049C, YER123W, YDR444W, YDR443C, YDR429C, YML115C, YDR432W, YDL119C, YDR120C, YGL151W, YBR159W, YBR095C, YBR086C, YMR101C, YHR171W, YBR087W, YDR405W, YBR110W, YBR120C, YIL127C, YBR101C, YBR098W, YBR109C, YBR102C, YBR082C, YBR056W, YBR053C, YBR059C, YBR057C, YBR042C, YBR040W, YML070W, YBR044C, YMR093W, YGL183C, YBR080C, YBR079C, YBR061C, YBR060C, YBR264C, YBR065C, YPL266W, YGR090W, YBR187W, YIL026C, YGL160W, YBR163W, YBR170C, YBR168W, YBR200W, YNL274C, YBR220C, YNL187W, YBR193C, YHR076W, YBR196C, YBR195C, YBR162C, YBR132C, YPL190C, YBR135W, YMR064W, YBR123C, YBR121C, YBR128C, YBR125C, YBR155W, YBR154C, YBL014C, YBR156C, YBR141C, YHR204W, YMR236W, YBR142W, YER157W, YAR015W, YAR008W, YAR019C, YAR018C, YGR103W, YER065C, YAR007C, YAR003W, YMR301C, YBR160W, YDL033C, YBL016W, YBL007C, YKL189W, YBL011W,

YBL008W, YDR221W, YAL011W, YOR223W, YAL022C, YAL021C, YOR127W, YBR074W, YAL009W, YER164W, YGL122C, YAL038W, YAL044C, YLR222C, YAL027W, YDR195W, YAL034W-A, YAL031C, YBL105C, YPR163C, YBR002C, YBL107C, YGR080W, YBL091C, YBL103C, YBL095W, YBR029C, YKR016W, YHR025W, YBR030W, YBR011C, YLR006C, YBR025C, YNL255C, YBL090W, YCL054W, YGR009C, YLR170C, YBR151W, YBL028C, YKL114C, YBL032W, YGL171W, YOL122C, YDR264C, YBL078C, YBL076C, YBL057C, YJL148W, YPR187W, YBL061C, YDL164C, YDL046W, YDL044C, YOR118W, YDL047W, YDL031W, YDL025C, YDR364C, YBL020W, YDL072C, YDL065C, YDR356W, YLR226W, YDL056W, YDR238C, YDL064W, YKR067W, YDL018C, YCR084C, YCR082W, YPL046C, YCR086W, YOL001W, YPL148C, YOR330C, YKL143W, YDL004W, YFR052W, YDL014W, YDL013W, YCR092C, YCR088W, YCR095C, YOR251C, YDL128W, YOR274W, YDL133W, YBR192W, YNL201C, YDL115C, YDR430C, YPR037C, YDL153C, YDL150W, YDL160C, YNL221C, YDL139C, YDL135C, YDL143W, YLR075W, YOL096C, YDL097C, YDL092W, YDL100C, YDL087C, YLR457C, YJL112W, YGR233C, YDL098C, YDL110C, YDL108W, YMR311C, YBR243C, YDL102W, YNL100W, YDL107W, YIL033C, YCR065W, YBR275C, YMR092C, YBR279W, YBR276C, YBR265W, YBR070C, YGR072W, YBR267W, YER142C, YBR290W, YLR452C, YCL001W, YBR283C, YBR282W, YOL136C, YHL013C, YBR261C, YBR237W, YBR236C, YKL193C, YNL186W, YOR254C, YIR006C, YBR234C, YBR233W, YIL008W, YBR254C, YBR260C, YBR258C, YBR248C, YBR247C, YBR252W, YBR251W, YCR028C-A, YCR028C, YLL023C, YCR032W, YDR068W, YCL061C, YCR011C, YGR128C, YCR053W, YCR052W, YOR073W, YOR336W, YCR042C, YCR036W, YCR051W, YNL277W, YCL059C, YCL027W, YBL104C, YCL031C, YOR046C, YCL014W, YCL009C, YBR173C, YCL016C, YCL052C, YMR295C, YCL055W, YBL035C, YCL034W, YCL032W, YIL065C, YFL002C, YGR089W, YBR171W, YGR091W, YGR094W, YIL090W, YGR081C, YBR041W, YJR062C, YFR005C, YGR104C, YER041W, YLL034C, YGR250C, YMR013C, YGR099W, YGR102C, YKL046C, YGR031W, YGR040W, YGR046W, YGL154C, YOL022C, YGR013W, YGR019W, YBR269C, YGR075C, YGR076C, YGR077C, YDR321W, YGR057C, YGR058W, YGR061C, YGR170W, YHR088W, YGR172C, YDR306C, YDR419W, YGR156W, YGR163W, YPR129W, YLR419W, YBR049C, YGR185C, YOR036W, YGR174C, YGR175C, YGR178C, YER078C, YGR112W, YGR119C, YGR120C, YGR122W, YDR190C, YGR117C, YGR113W, YGR116W, YGR144W, YGR149W, YGR150C, YGR152C, YOR259C, YGR127W, YHR170W, YHR034C, YGL155W, YJL084C, YGL163C, YGL141W, YGL142C, YGL173C, YGL153W, YGL143C, YGL174W, YOR232W, YGL180W, YKL064W, YGL169W, YJR040W, YGL172W, YGL116W, YGL119W, YGL120C, YOR372C, YPR054W, YPR116W, YGL113W, YOR212W, YGL136C, YNL216W, YOR216C, YGL140C, YGL123W, YGL125W, YGL129C, YOR245C, YGL240W, YGL241W, YOL098C, YGL248W, YDR179W-A, YGL234W, YER162C, YGL238W, YHL019C, YGR005C, YGR006W, YGR007W, YEL058W, YML094W, YGR001C, YGR003W, YGL194C, YOR266W, YGL200C, YGL201C, YGL181W, YBR073W, YGL190C, YGL192W, YLR054C, YGL221C, YGL223C, YOR174W, YGL202W, YGL016W, YGL207W, YGL212W, YHR101C, YDR232W, YHR109W, YER156C, YDL132W, YHR078W, YDR369C, YGR171C, YHR119W, YHR120W, YHR121W, YOR074C, YHR111W, YDL116W, YHR114W, YKR064W, YHR042W, YOR119C, YHR049W, YHR052W, YGR240C, YHR032W, YHR036W, YHR038W,

YHR151C, YPL160W, YHR073W, YHR075C, YHR058C, YJL008C, YEL048C, YHR061C, YHR178W, YHR181W, YHR187W, YIR001C, YCR008W, YBR088C, YHR172W, YBR026C, YHR196W, YHR197W, YHR199C, YNL330C, YHR191C, YHR192W, YHR193C, YLR259C, YOR367W, YHR142W, YHR147C, YHR148W, YHR127W, YHR132C, YPL093W, YHR134W, YGL114W, YHR167W, YHR168W, YHR169W, YHR150W, YKL207W, YHR159W, YHR164C, YGL245W, YNL222W, YGR246C, YDL088C, YBR003W, YGR237C, YGR239C, YGR258C, YGR261C, YGR262C, YKR038C, YGR095C, YKL014C, YGR252W, YGR253C, YGR196C, YGR198W, YDR234W, YGR205W, YJR140C, YGR193C, YPR025C, YNL163C, YGR225W, YER147C, YGR231C, YGR232W, YGR208W, YGR210C, YGR215W, YGR218W, YHR004C, YHR005C, YHR007C, YNL137C, YHL030W, YOL041C, YHL032C, YBR291C, YHR024C, YBR034C, YIL150C, YHR031C, YHR011W, YHR013C, YIL010W, YHR018C, YGR276C, YGR284C, YJR132W, YHL004W, YGR264C, YGR270W, YGR272C, YOL095C, YGR181W, YHL022C, YHL023C, YHL024W, YLR208W, YHL014C, YKL095W, YGR179C, YAL059W, YMR005W, YFR008W, YJR105W, YFL046W, YJR072C, YFR021W, YFR028C, YPR040W, YPR073C, YFR010W, YOL030W, YFL027C, YFL028C, YFL029C, YFL013C, YFL016C, YFL023W, YFL038C, YIL094C, YDR448W, YHR102W, YFL034C-B, YFL037W, YOR334W, YJL122W, YGL017W, YGL005C, YGL011C, YGL012W, YGL023C, YFR043C, YGL027C, YGL018C, YOR065W, YGL022W, YFR041C, YGL026C, YFR045W, YMR270C, YNR018W, YDR196C, YDL001W, YGL001C, YFL044C, YLR219W, YFR049W, YFR050C, YER112W, YER118C, YER124C, YJR063W, YER110C, YCL029C, YER133W, YER136W, YER178W, YER125W, YER126C, YER009W, YER083C, YHR059W, YHR060W, YER080W, YER134C, YER082C, YER093C-A, YER095W, YER100W, YER090W, YER091C, YDR376W, YDR006C, YER182W, YER183C, YER168C, YJR101W, YEL032W, YNR011C, YNL113W, YDR240C, YFL001W, YMR100W, YNL241C, YGR229C, YNL223W, YER149C, YER141W, YHL039W, YER143W, YJL069C, YGL236C, YER165W, YER155C, YHR110W, YBR038W, YHL016C, YML088W, YHR008C, YKL034W, YGL079W, YGL078C, YGL077C, YGL059W, YGL048C, YBR249C, YGL040C, YGR166W, YGL055W, YMR160W, YLR078C, YGL098W, YGL097W, YGL110C, YGL106W, YGL103W, YPL023C, YMR127C, YGL085W, YCR066W, YLR440C, YGL091C, YMR010W, YGL036W, YKL205W, YGL029W

T12b:

YPR131C, YFR009W, YLR323C, YFL045C, YBL057C, YDL147W, YLR275W, YLR277C, YJL074C, YFL002C, YML096W, YDR454C, YFL022C, YLR409C, YJR068W, YLR005W, YLR208W, YGL137W, YLR212C, YGL130W, YLR086W, YGL211W, YGL201C, YPR066W, YLR243W, YGL095C, YBR236C, YGL043W, YGR185C, YGL112C, YLR234W, YLL011W, YNL222W, YAL035W, YOR006C, YER007C-A, YHR122W, YNL207W, YDR460W, YIL068C, YOR216C, YDR339C, YGR264C, YNL240C, YJL050W, YNL247W, YNL250W, YMR167W, YBL035C, YMR236W, YBR234C, YJL033W, YNL263C, YPR036W, YMR131C, YDR267C, YNL102W, YMR240C, YNL113W, YNL061W, YNL072W, YNL262W, YNL082W, YIL021W, YIL003W, YKL078W, YOL097C, YLR060W, YKL045W, YDR196C, YHR111W, YLR115W, YHR070W, YGR172C, YMR093W, YHR169W, YKL095W, YFL008W, YJR017C, YOL094C, YIR022W, YIR008C, YJL097W, YJL115W, YOL005C, YJL014W, YGL207W, YIL103W,

YDR140W, YKL028W, YHR148W, YJR072C, YIL143C, YBL019W, YML021C, YNL132W, YDR167W, YLL034C, YBR221C, YLR002C, YGR173W, YGR020C, YGR005C, YNL312W, YGL221C, YCL059C, YML080W, YKL190W, YGR285C, YDL132W, YKL151C, YKR038C, YKR079C, YHR069C, YHR068W, YKL213C, YHR020W, YBR058C, YGR246C, YGR195W, YGR200C, YMR128W, YGL099W, YDR331W, YLR383W, YOR262W, YJL141C, YOR095C, YPR176C, YNL288W, YGR125W, YNL088W, YPL029W, YJL091C, YBR170C, YOR291W, YLR347C, YOR116C, YBR196C, YOR091W, YPL266W, YPL022W, YDL030W, YDL140C, YOL021C, YDL108W, YLR195C, YDL028C, YOR157C, YDL001W, YOR119C, YDL064W, YMR061W, YPL160W, YML102W, YMR235C, YPR107C, YMR092C, YBL076C, YPR056W, YHR019C, YPR082C, YPR161C, YCR057C, YPR183W, YBL023C, YPR112C, YPR113W, YMR190C, YCR042C, YPL151C, YJR006W, YCL004W, YDR172W, YDL102W, YPL122C, YPR048W, YPL008W, YOR048C, YPR016C, YNL022C, YBR061C, YPL175W, YPL217C, YNR043W, YDR376W, YGL106W, YDR189W, YDR050C, YDR037W, YLR447C, YNL317W, YOR117W, YDR212W, YDR238C, YER082C, YIL078W, YOL133W, YMR080C, YDR097C, YGL169W, YDR325W, YNR053C, YDR083W, YJL121C, YOL090W, YDR002W, YNL107W, YDR448W, YDL201W, YDL164C, YOR007C, YOL142W, YBR160W, YDR292C, YPL094C, YDR449C
